# LAMC1-mediated preadipocytes differentiation promoted peritoneum pre-metastatic niche formation and gastric cancer metastasis

**DOI:** 10.7150/ijbs.70524

**Published:** 2022-04-24

**Authors:** Yan Fang, Rongzhang Dou, Sihao Huang, Lei Han, Hang Fu, Chaogang Yang, Jialin Song, Jinsen Zheng, Xinyao Zhang, Keshu Liu, Zhenxian Xiang, Xinghua Zhang, Shuyi Wang, Bin Xiong

**Affiliations:** 1 Department of Gastrointestinal Surgery & Department of Gastric and Colorectal Surgical Oncology, Zhongnan Hospital of Wuhan University, Wuhan, Hubei, 430071, China.; 2 Department of obstetrics and gynecology, Guangzhou Women and Children's Medical Center, Guangzhou, Guangdong, 510623, China.; 3 Hubei Key Laboratory of Tumor Biological Behaviors, Wuhan, Hubei, 430071, China.; 4 College of Life Sciences of Wuhan University, Wuhan, Hubei, 430072, China.

**Keywords:** LAMC1, Gastric cancer peritoneal metastasis, Preadipocyte, Free fatty acids (FFAs), Predictive value

## Abstract

Gastric cancer is anatomically proximal to peritoneum. Gastric cancer peritoneal metastasis is a complex biological process which is corresponded with disharmony within dysfunctional adipose tissue and metabolism reprogramming. Laminin gamma 1 (LAMC1) is highly expressed in cancer cells of peritoneal metastatic sites, however, the mechanism of LAMC1-metiated gastric cancer metastases to adipose tissue-rich peritoneum remains unclear. In our study, immunohistochemical staining, single cell sequencing, a co-culture model, luciferase reporter, RNA immunoprecipitation (RIP), Chromatin immunoprecipitation (CHIP) and single-molecular magnetic tweezers assays were conducted, and our results showed that LAMC1 related to Perilipin-1 content was highly expressed in peritoneal metastatic sites and mainly secreted by tumor cells. Gastric cancer cells secreted LAMC1 in an autocrine manner to detached from the primary site and promoted preadipocytes mature, rupture and release of free fatty acids (FFAs) in the peritoneal microenvironment to form pre-metastatic niche by the paracrine pathway. Reversely, differentiated preadipocyte-derived conditioned medium inhibited glycolysis and enhanced fatty acid oxidation (FAO) rate to promote cell proliferation, mesenchymal-epithelial transformation which led to tumor peritoneal colonization. In terms of biological mechanisms, one of differentiated preadipocyte-derived FFAs, palmitic acid-activated STAT3 inhibited miR-193a-3p by binding to its promoter directly; Using single-molecular magnetic tweezers, this binding manner was proved to be stable, reversable and ATP-dependent. Moreover, miR-193a-3p regulated LAMC1 in a post-translational manner. Furthermore, high LAMC1 expression in serum predicted a higher risk of peritoneal metastasis. In conclusion, our results illustrated that palmitic acid/p-STAT3/miR-193a-3p/LAMC1 pathway promotes preadipocyte differentiation, pre-metastatic niche formation and gastric cancer cell colonization to peritoneum.

## Introduction

Metastasis is main cause of cancer-related death in patients with gastric cancer. Liver, lung, bone and peritoneum are all friendly sites for gastric cancer cells to grow and form metastatic lesions 1, 2. Peritoneal metastases of gastric cancer are associated with significantly poor prognosis and high mortality^3^. According to Stephen Paget's 'seed and soil' hypothesis, the peritoneal proclivity of gastric cancer metastasis depends on the interaction of tumor cells and peritoneal microenvironment 4, 5, therefore it's necessary to explore what the roles that microenvironment plays in gastric cancer metastasis.

Peritoneal microenvironment contains extracellular matrix (ECM), cells and some secretory proteins. Adipocytes, derived from preadipocytes, are major cell components in the peritoneum^6^, ^7^ and beneficial to tumor peritoneal implantation. On the one hand, adipocytes and tumor cells detached from the primary site have a reciprocal interplay which enhances the ability of migration, invasion and homing of tumor cells^8^, ^9^; On the other hand, adipocytes reprogram tumor metabolism leading to rapid metastatic growth via fatty acids oxidation (FAO). In a gastric cancer omentum implant model, adipocytes release large amounts of fatty acids to provide adequate energy for adjacent gastric cancer cells which further increase their invasive capacity^10^. The differentiation of preadipocyte to adipocyte was highly correlated with ECM remodeling and within this process, some proteins played a significant role 11. Our previous mass spectrometry results showed that compared with the primary tumor, LAMC1 was highly expressed in gastric cancer peritoneal metastases and GO enrichment analysis revealed that LAMC1 was involved in ECM organization, disassembly and substrate adhesion-dependent cell spreading. However, how LAMC1 affects the peritoneal metastasis process remains unclear.

LAMC1 encoding the laminin subunit gamma 1 protein is involved in the metastatic process of multiple cancers^12^, ^13^. Moreover, LAMC1 may also participate in the regulation of adipogenesis^14^. By binding with integrin α6 in preadipocyte membrane, LAMC1 facilitates the conversion of nonfunctional preadipocytes into adipocytes, which had secretory capacity of adipokine and fatty acids 15-17. Besides, LAMC1 is also related to ECM remodeling and elevated tissue stiffness. The mechanical shear produced by the rigid ECM can accelerate breakdown of mature adipocytes and release of free fatty acids (FFAs) which provide more energy for adjacent gastric cancer cells^18^. Therefore, we postulate that gastric cancer cells can rewire their biology to secrete more LAMC1 to act on preadipocytes, promote lipid formation and peritoneal metastasis, and yet the mechanisms of interaction have not been fully elucidated.

Given the biological and physical functions of LAMC1, we speculate that gastric cancer cells secrete LAMC1 to promote their abscission from the primary site and promote preadipocyte maturation to form pre-metastatic niche formation in peritoneal microenvironment. At present, our results show that LAMC1 is highly expressed in gastric cancer and mainly secreted by gastric cancer cell which is associated with poor prognosis. *In vitro* co-culture experiments have shown that gastric cancer cells secrete LAMC1 protein to enhance mobility by autocrine way and adipogenesis in a paracrine manner. Moreover, differentiated preadipocytes induce a partial mesenchymal-epithelial transition (MET) of adjacent tumor cells to form metastasis and adipocyte-derived conditioned medium (CM) inhibited glycolysis and promoted fatty acid oxidation by remodeling metabolic programming. Further mechanistic studies revealed that gastric cancer cells orchestrate palmitic acid/p-STAT3/miR-193a-3p/LAMC1 axis to support more LAMC1 secretion, which further favors preadipocyte maturation to consolidate pre-metastatic niche. Moreover, high LAMC1 expression in serum was related to high risk of peritoneal metastasis. These findings demonstrate dynamic changes of adipose tissue in peritoneal microenvironment and a positive feedback loop between gastric cancer cells and preadipocytes, which contributes to our understanding molecular of the mechanism for peritoneal metastasis of gastric cancer, meanwhile, LAMC1 had good predictive value for peritoneal metastasis.

## Material and methods

### Patient tissue samples

In this study, we collected tissue samples from 90 patients who had been diagnosed with gastric cancer by histopathological assessment and underwent curative resection (Table S1) and blood from 77 patients. Another 3 paired tissue samples were obtained from gastric cancer peritoneal metastasis site, primary site and normal tissue adjacent to cancer at the Zhongnan Hospital of Wuhan University (Table S2). Prior to surgical resection, the patient had not received any chemotherapy or radiotherapy. All samples were obtained with informed patients' consent before collection, and approved by the Zhongnan Hospital of Wuhan University Ethics Committee. Moreover, the collected samples were fixed in formalin and embedded in paraffin for further study.

### HE staining, immunohistochemistry, immunofluorescence

A section of paraffin-embedded peritoneal samples and primary focus were taken for HE staining. The antibodies used in immunohistochemistry as follows: LAMC1 (PAB32791; 1:1000; Bioswamp, China), Perilipin-1 (9349; 1:200; Cell Signaling, USA), LDHA (66287-1-Ig; 1:100; Proteintech, USA), CPT1B (22170-1-AP; 1:100; Proteintech, USA), FABP4 (67167-1-Ig; 1:500; Proteintech, USA), CD36 (66395-1-Ig; 1:500; Proteintech, USA; 1:500; Proteintech, USA) and Ki67 (ab16667; 1:250; Abcam, USA). The antibodies used in immunofluorescence as follows: LAMC1 (PAB32791; 1:100; Bioswamp, China), Ki67 (ab16667; 1:250; Abcam, USA) and Perilipin-1 (9349; 1:200; Cell Signaling, USA). The expression levels were evaluated by mean density (Integrated optical density / Pixel area of the tissue) under 200× magnification.

### Liquid chromatography mass spectrometry and mass spectrometry (LC-MS/MS)

We used Thermo Scientific's Tandem Mass Tag™ (TMT™) technology to label different protein samples and performed high-throughput LC-MS/MS quantitative research at the same time. The total protein was extracted from 4 samples, and the same amount of protein was taken to digest with Trypsin to obtain the peptides of the corresponding sample; Subsequently the 4 samples were labeled with different TMT tags and subjected to LC-MS/MS analysis. For cell mass spectrometry, label-free quantification was used.

### Analysis of single-cell sequencing data

The tumor tissue dataset^19^ was extracted and processed into standard data using the seurat package (v4.0.2), then integrated using the harmony package (v0.1.0). Cell annotation was performed based on marker genes of various cell types to identify epithelial cells, fibroblasts, endothelial cells, immune cells. Then inferCNV package (v1.6.0) was used to distinguish tumor cells and non-tumor cells in extracted epithelial cells, fibroblasts and endothelial cells. The expression of target genes was visualized using the featureplot functions.

### Cell culture, human primary omental preadipocytes extraction and adipocyte differentiation induction

The human gastric cancer cell lines (AGS, BGC823, HGC27, SGC7901) cultured in RPMI 1640 medium (Gibco, USA), normal gastric epithelium cells line (GES-1) and mouse preadipocyte cell line (3T3-L1) cultured in DMEM medium (Gibco, USA) were purchased from Chinese Academy of Sciences in Shanghai. Human primary omental preadipocytes were extracted according to protocol^20^. For 3T3-L1 and human preadipocytes induction, the medium of cells was cultivated with DMEM containing 10% FBS, 10ug/ml insulin, 1umol/L dexamethasone, 0.5mmol/L 3-isobutyl-1-methylaxanthine (IBMX, HY-12318; MCE) for 2 days after 2 days of contact inhibition, and subsequently, the medium was replaced with high glucose DMEM medium containing 10ug/ml insulin and 10% FBS for 2 days, then, change the medium every day with DMEM supplemented with 10% FBS until 3T3-L1 and human preadipocytes were induced into mature adipocyte^21^. In order to confirm the effect of LAMC1 on the differentiation of adipocytes, different concentrations of LAMC1 recombinant protein (0, 50ng/ml, 100ng/ml) (Ag14674; Proteintech, USA) dissolved in phosphate-buffered saline (PBS, pH 7.4) were added to each fresh medium used for replacement.

### Transfections with siRNAs, miRNAs

Hsa-miR-193a-3p mimic, inhibitor, negative control (NC) and STAT3 siRNAs (NC, si-1 and si-2) from RiboBio (Guangzhou, China), LAMC1 siRNAs (NC, si-1, si-2 and si-3) from GenePharma (Shanghai, China) and lipofectamine 2000 (Invitrogen, USA) were purchased for transfection according to the manufacturer's protocol.

### Oil Red O staining

The 6-well plate was fixed with 4% paraformaldehyde for 30 minutes and stained with Oil Red O for 10 minutes.

### RNA isolation and quantitative real-time PCR (RT-qPCR)

According to the manufacturer's instructions, the Trizol Reagent was used to isolate the total RNA from gastric cancer cells, adipocytes or tissues. Measure RNA concentration and quantify 1ug RNA for reverse transcription using the PrimeScript^TM^ RT reagent kit (Toyobo, Osaka), then RT-qPCR was run on the Bio-Rad IQ5 Real PCR machine (Bio-Rad, USA) in a 96-well plate containing reaction mixture of pre-primer, post-primer, cDNA, SYBR-Green PCR Master Mix (Takara, Osaka). We used the 2^-ΔΔCt^ method to calculate the relative expression. The primer sequences used in the study are presented in Table S3.

### Western blot

Cells or tissues were lysed in RIPA buffer, including a protein-phosphatase inhibitor cocktail (Thermo Scientific, USA) at 4℃, then use 10% SDS-PAGE gels to separate the total proteins before they are transferred electrophoretically (Bio-Rad, USA) onto PVDF membranes (Millipore, USA). Bio-Rad ChemiDoc XRS +System was used to detect the protein after dropping the developer solution (Bio-Rad, USA) onto the blots. The primary antibodies were purchased in this study as follows: anti-LAMC1 (PAB32791; 1:1000; Bioswamp, China), anti- CEBPα (18311-1-AP; 1:1000; Proteintech, USA), anti- PPARγ (16643-1-AP; 1:1000; Proteintech, USA), anti-DLK1 (10636-1-AP; 1:1000; Proteintech, USA), anti-HSL (17333-1-AP; 1:1000; Proteintech, USA), anti-Perilipin-1 (9349; 1:1000; Cell Signaling, USA), anti-MEK1/2 (11049-1-AP; 1:1000; Proteintech, USA), anti-p-MEK1/2 (phosphor Ser217/221) (3958; 1:1000; Cell Signaling, USA), anti-ERK1/2 (16433-1-AP; 1:1000; Proteintech, USA), anti-p-ERK1/2 (phosphor Thr202/Tyr204) (AF1015; 1:1000; Affinity Biosciences, USA), anti-STAT3 (10253-2-AP; 1:1000; Proteintech, USA), anti-p-STAT3 (phosphor Tyr705) (9145; 1:1000; Cell Signaling, USA), mTOR (2983T; 1:1000; Cell Signaling, USA), anti-p-mTOR (phosphor Ser2448) (5536T; 2983T; 1:1000; Cell Signaling, USA), anti-GAPDH (sc-32233; 1:5000; Santa Cruz, CA), and anti-β-tubulin (sc-5274; Santa Cruz, CA).

### Agarose gel electrophoresis and transmission electron microscope

DNA samples were separated with 0.5% agarose gel. Bio-Rad ChemiDoc XRS +System was used to observe the gel and photograph. The electron microscopy samples were observed under the transmission electron microscope (TEM, HT7700, Hitachi).

### NADPH, NADP^+^ and ATP assay

The cells in the six-well plate were collected into EP tube, and the contents of total NADP (NADPH and NADP^+^) in the cells were determined according to the methodology of NADP^+^/NADPH detection kit (S0179, Beyotime, China). The concentration of NADP^+^ was calculated as follows: [NADP^+^] = [NADP_total_] - [NADPH]. The ATP detection was performed according to protocol of ATP assay kit (A095-1-1, Nanjing Jiancheng Bioengineering Institute, China). All experiments were performed in triplicate and measured accurately with unit protein amount.

### Glycolysis, fatty acid oxidation and extracellular O_2_ consumption assay

The kits were purchased from Abcam. Glycolysis kit (ab197244, Abcam, USA), fatty acid oxidation kit (ab217602, Abcam, USA) and extracellular O2 consumption kit (ab197243, Abcam, USA) were used for analysis of glucose and lipid metabolism according to protocol.

### Enzyme-linked immunosorbent assay (ELISA)

ELISA kit (Bioswamp, China) was used to measure the content of LAMC1, FFAs, adiponectin, leptin and Perilipin-1 in the supernatant according to manufacturer's protocol.

### Luciferase reporter assay

The miRNA Dual-Luciferase target expression vector pmirGLO (Promega, USA) is designed to evaluate miRNA activity quantitatively by inserting miRNA target sites 3'-UTR sequences of LAMC1. Lipofectamine 2000 was used to transfected miRNA mimics, inhibitor or negative control and constructed vector into 293T cells to perform a luciferase experiment. For the miR-193a-3p promoter assay, a 2000-bp and its truncation (-509/0, -765/0) containing STAT3 binding sites were inserted into the vector PGL-4.1 (Promega, USA). Genomic STAT3 was inserted into vector PCDNA3.1 (Promega, USA). Renilla luciferase vector pRL-SV40 (Promega, USA) acted as an internal reference for normalization and selection. Dual-Luciferase Reporter Assay System (Promega, USA) was used to detect luciferase.

### RNA immunoprecipitation (RIP) assay and Chromatin immunoprecipitation (ChIP) assay

RIP was performed using the Magna RIP RNA-Binding Protein Immunoprecipitation Kit (Millipore, MA, USA) and ChIP assays were performed using a SimpleChIP® En-zymatic Chromatin IP Kit (Cell Signaling, #9003, USA) according to the manufacturer's protocol. The final precipitated DNA specimens were amplified a 174bp region (CHIP1) with the primers TGAGGCTGGGGATCTTGGTA (forward) and GTGGAATCCCCTTGGTGGAG (reverse) and a 256bp region (CHIP2) with the primers CCAAGGGGATTCCACTGACC (forward) and GCCTGCAGACAACCTCTCTT (reverse). The negative control was GAPDH with the primers TCGAACAGGAGGAGCAGAGAGCGA (forward) and TACTAGCGGTTTTACGGGCG (reverse).

### Single-molecule magnetic-tweezers

Protein-DNA interactions could be quantified and characterized by single-molecule magnetic-tweezers^22^. The reaction mixture was composed of 100μM ATP, 20mM Tris-HCl, 50mM MgCl2, 0.1%-0.2% BSA, 5nM STAT3 recombinant protein (Active motif, USA), 5nM JAK2 purified protein (Abcam, USA) and promoter region of miR-193a-3p containing 2000bp. All samples were incubated at 37°C for 10 min, and the principle of single-molecule magnetic-tweezers could refer to literature^23^.

### Animal assays

All BALB/c athymic nude mice (female, 4 weeks old and 16-20g) were purchased from Hubei Research Center of Laboratory Animals (Wuhan, China) and all experimental operations were approved by the animal ethics committee of Zhongnan hospital of Wuhan University. For the gastric cancer peritoneal metastasis model, the nude mice were divided into 2 groups (n=5 per group). AGS cells (1×10^7) transfected with LAMC1 NC or siLAMC1 lentiviral vector were intraperitoneally injected into nude mice. For the xenograft model to mimic primary site of gastric cancer, the nude mice were divided into 2 groups (n=5 per group). AGS cells (1×10^7) transfected with LAMC1 NC or siLAMC1 lentiviral vector were subcutaneously injected into right flank of nude mice. For the xenograft model to mimic status before peritoneum implantation, the nude mice were divided into 2 groups (n=5 per group). A cell mixture of AGS cells (1×10^7) transfected with LAMC1 NC or siLAMC1 lentiviral vector and 3T3-L1 (5×10^4) in 200ul were subcutaneously injected into right flank of nude mice. After 30 days, the nude mice were sacrificed for further HE and immunohistochemistry evaluation. The tumor burden was assessed by tumor volume (1/2×width^2^ × length) and weight.

### Statistics analysis

Results expressed as mean ± standard error (m ± SE) were analyzed by two-tailed Student's t-test. All experiments were performed in triplicate and statistical significance was defined as p < 0.05 using the Graph- Pad Prism software (version 6.0, GraphPad Software, USA).

## Results

### Gastric cancer has a high expression of LAMC1 and are prone to colonize to adipocyte-rich peritoneum

**Figure 1:**
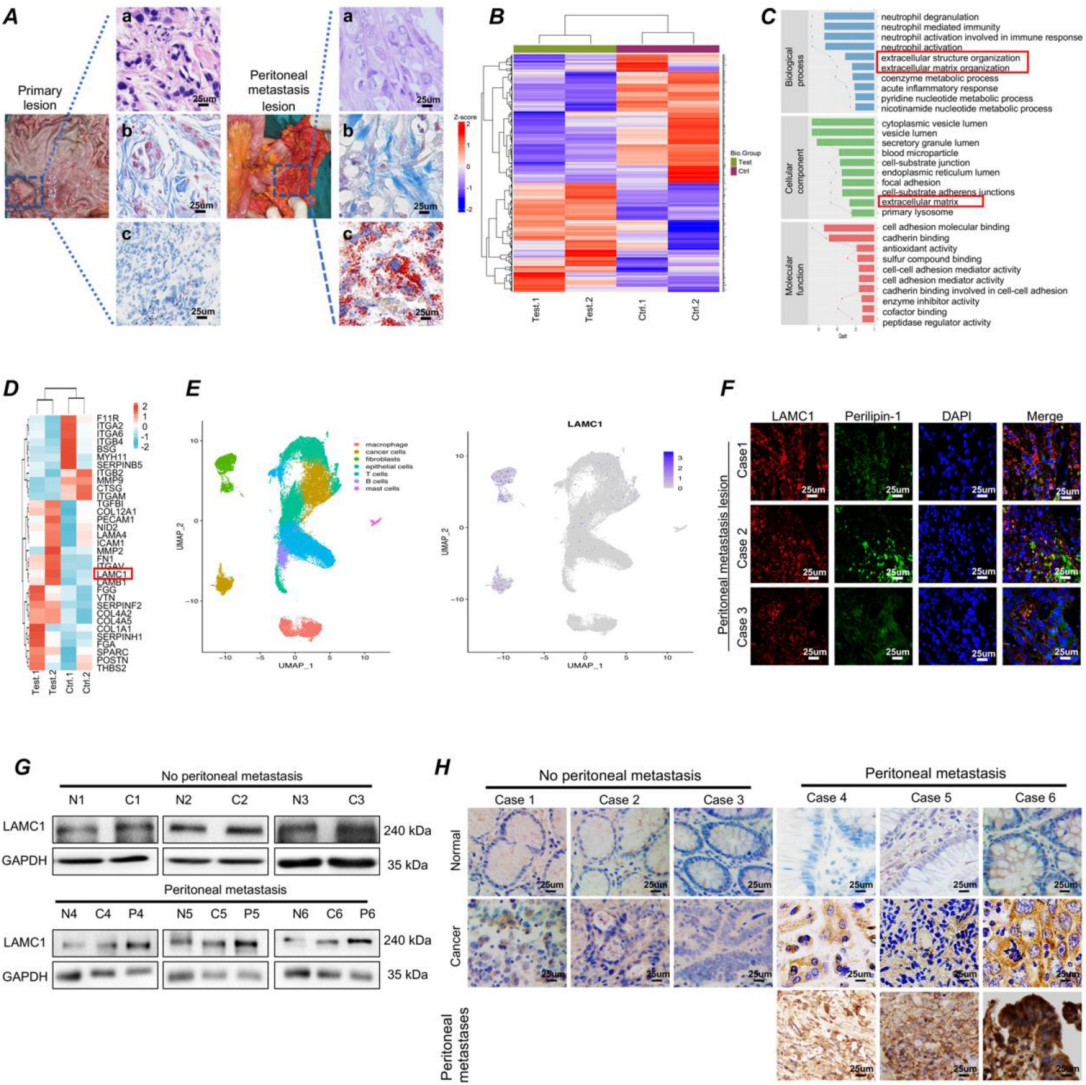
Gastric cancer has a high expression of LAMC1 and are prone to colonize to adipocyte-rich peritoneum. **(A)** (a) HE (b) Masson (c) Oil Red O staining results of primary lesion and peritoneal metastasis lesion were shown. **(B)** The clustering heatmap of significant proteins.** (C)** Gene function classification. **(D)** Clustering heatmap of extracellular matrix organization proteins. **(E)** Analysis of single-cell sequencing data. **(F)** The correlation between LAMC1 and Perilipin-1 was analyzed by immunofluorescence staining in peritoneal metastasis samples. **(G)** Western blots result for analyzing LAMC1 expression in patients' tissue specimen with or without peritoneal metastasis.** (H)** Immunohistochemistry for analyzing LAMC1 expression in patients' tissue specimen with or without peritoneal metastasis.

In our long-term clinical work, we had found that gastric cancer had a predisposition to metastasize to the peritoneum. Masson staining showed fibrosis occurred at the metastatic site and the Oil Red O staining showed more neutral lipid droplets were present at the metastatic site compared with the primary lesion (Fig. 1A). Mass spectrometry results of two paired gastric cancer peritoneal metastatic cancer samples demonstrated that compared with primary lesion, the differential proteins were involved in extracellular structure and matrix organization (Fig. 1B-1C). Among extracellular matrix organization proteins, SPARC, COL1A1, FN1 and LAMC1 had a high expression, cell experiments showed LAMC1 was highly expressed in all gastric cancer cell lines (Figure S1A), and mass spectrometry results indicated that the expression of peritoneal metastases was 1.59 times higher than that in primary foci (Fig. 1D). Furthermore, according to single cell sequencing data of gastric cancer, LAMC1 was mainly from fibroblasts and cancer cells (Fig.1E). The number of cancer cells was much higher than fibroblasts in tumor environment, so tumor cells were considered as the primary source of LAMC1. Immunofluorescence analysis of peritoneal metastases in human specimens showed that regions with high expression of LAMC1 were accompanied by high expression of Perilipin-1, a lipid droplet coating protein (Fig. 1F). The Western blotting and immunohistochemical results showed that the expression level of LAMC1 was metastatic site > primary site > normal tissue and LAMC1 expression of primary site in patients with peritoneal metastasis was higher than that without peritoneal metastasis (Fig. 1G-1H). To further explore the role of LAMC1, we collected 90 specimens of gastric cancer for immunohistochemistry and LAMC1 expression was high in specimens whose average optical density is higher than the median. Our results showed that high LAMC1 expression was closely associated with T stage, TNM stage and peritoneal metastasis of gastric cancer (Table 1). We plotted a survival curve according to TCGA database and results showed that patients with high LAMC1 expression had a reduced overall survival and first progression survival (Figure S1B).

### Gastric cancer with high expression of LAMC1 have higher migration and invasion ability in primary site

**Figure 2:**
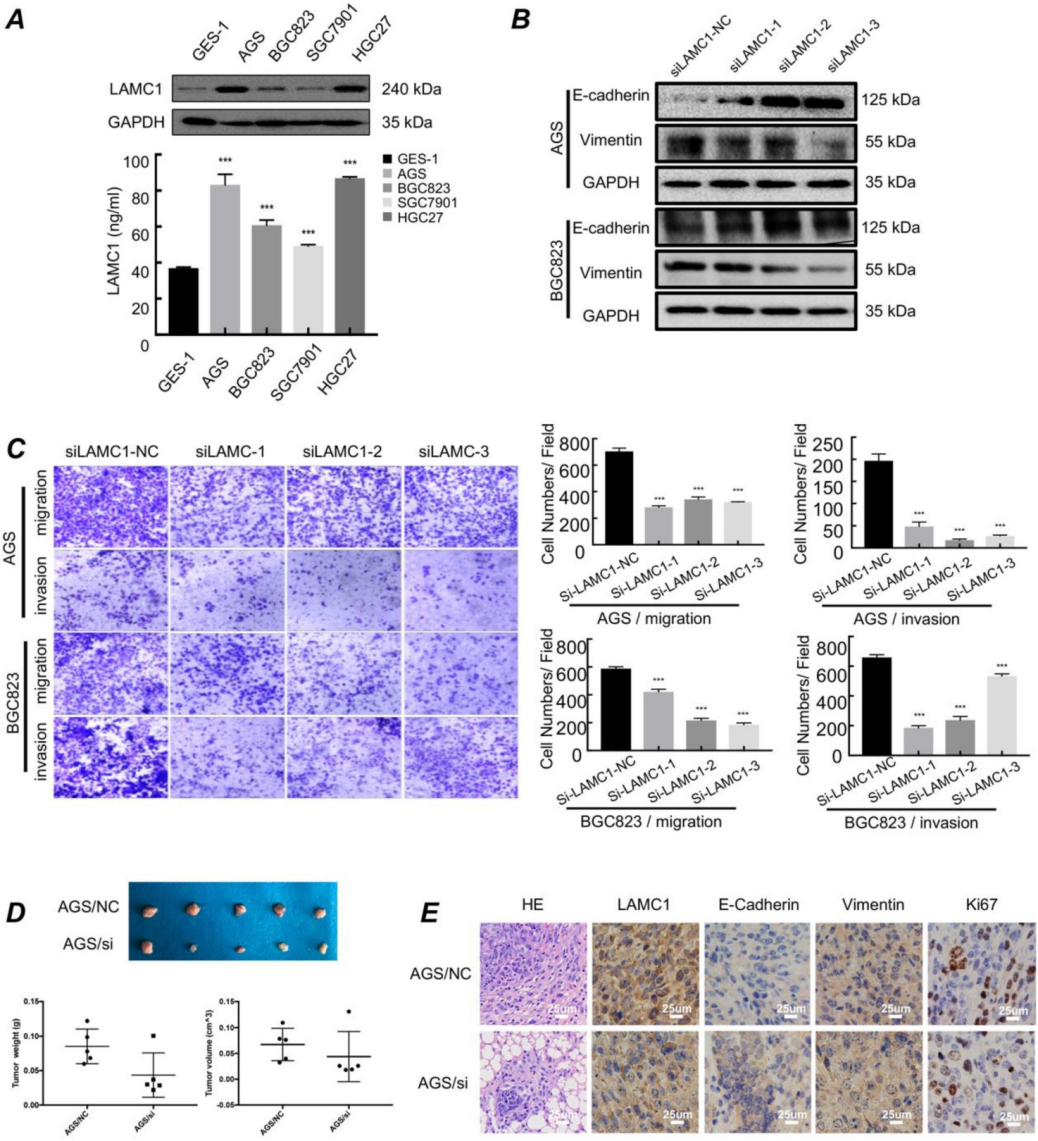
Gastric cancer with high expression of LAMC1 have higher migration and invasion ability in primary site. **(A)** Expression of LAMC1 in gastric cancer cell lines was analyzed by ELISA and Western blots. **(B)** Western blots were used for E-cadherin and vimentin expression in gastric cancer cells with LAMC1 knockout. **(C)** Transwell assay in gastric cancer cells with LAMC1 knockout.** (D)** The morphological characteristics of tumor xenograft model in AGS NC/si group, and tumor weight and volume were shown. **(E)** The HE staining and immunohistochemical results of xenograft tumor in AGS NC/si group.

We used western blots and ELISA to examine the expression of LAMC1 and the results showed that LAMC1 was up-regulated in cell lines and the conditioned medium of gastric cancer in protein level (Fig. 2A). AGS and BGC823 cell lines with LAMC1 knockout had a high expression of E-cadherin and low vimentin expression (Fig. 2B; Figure S1C-1D). Transwell assay results showed gastric cancer cells with stable LAMC1 expression acquired enhancive cell migratory and invasive ability than LAMC1 knockout groups (Fig. 2C). Cell scratch test confirmed that gastric cancer with LAMC1 knockout had lower wound healing ability (Figure S2A). We further mimicked the primary foci of gastric cancer using subcutaneous tumorigenesis model of nude mice to explore our results *in vivo* animal assay, and found that AGS cells with low LAMC1 expression developed smaller tumor size and body weight (Fig. 2D). Immunohistochemical staining results revealed that subcutaneous tumor tissue constructed by LAMC1 knockout cell line had up-regulated expression of E-cadherin, down-regulated vimentin and Ki67 expression (Fig. 2E).

### LAMC1-mediated preadipocytes differentiation promotes pre-metastatic niche formation and gastric cancer cell colonization in peritoneal microenvironment

**Figure 3:**
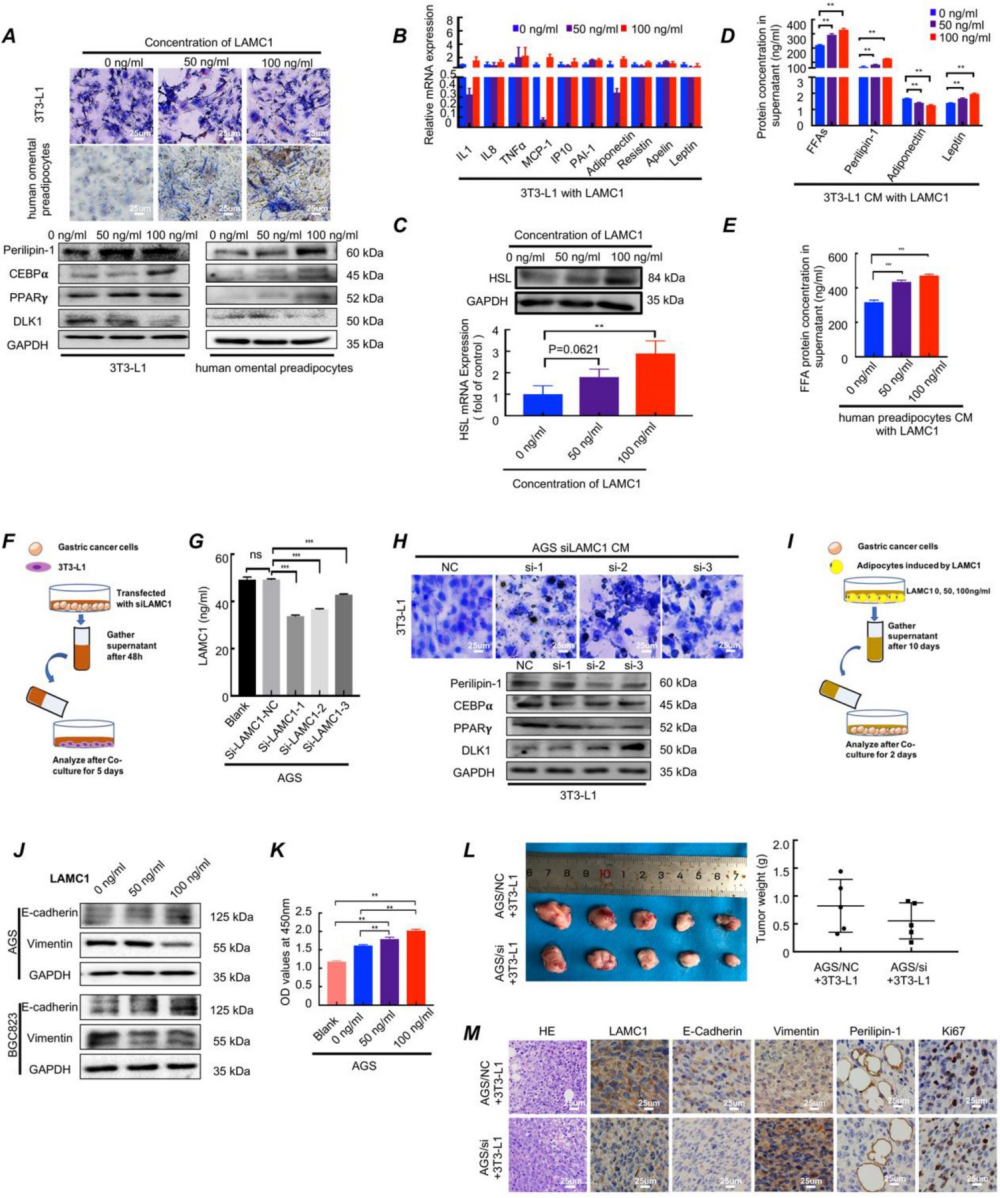
LAMC1-mediated preadipocytes differentiation promotes pre-metastatic niche formation and gastric cancer cell colonization in peritoneal microenvironment. **(A)** The Oil Red O staining and Western bolts for analyzing effect of different concentrations of LAMC1 (0, 50ng/ml and 100ng/ml) on 3T3-L1 and human omental preadipocytes differentiation for 5 days. **(B)** Cytokines secreted by 3T3-L1 after induction of different concentrations of LAMC1 for 24h were measured by RT-qPCR**.** (**C**) HSL expression in induced 3T3-L1 detected by RT-qPCR and western blots.** (D)** ELISA was used for analyzed FFAs, lipid droplets, adiponectin and leptin in 3T3-L1 CM after stimulation by different concentrations of LAMC1 for 4 days.** (E)** ELISA was used for analyzed FFAs in human omental preadipocytes CM after stimulation by different concentrations of LAMC1 for 4 days.** (F)** The flow chart of coculture. **(G)**ELISA for analyzing LAMC1 levels in supernatant of gastric cancer cell lines transfected with siLAMC1. **(H)** The lipid droplet formation ability of 3T3-L1 was measured by Oil Red O staining and adipocyte differentiation related protein after coculture for 5 days with gastric cancer cell supernatant transfected siLAMC1 for 48h.** (I)** Flowchart of reverse co-culture of 3T3-L1 supernatant and gastric cancer cells. **(J)** Western blots were used for E-cadherin and vimentin expression in gastric cells after coculture for 48h with 3T3-L1 supernatant induced by LAMC1 (0, 50ng/ml and 100ng/ml) for 4 days.** (K)** CCK8 assay for AGS cell proliferation detection**. (L)** The morphological characteristics of tumor xenograft model in AGS NC/si and 3T3-L1 coculture group, and tumor weight were shown. **(M)** The HE staining and immunohistochemical results of xenograft tumor in AGS NC/si and 3T3-L1 coculture group. Error bars, SD. *P < 0.05; **P < 0.01; ***P < 0.001.

To further explore the function of LAMC1, three concentrations of human LAMC1 recombinant protein (0 ng/ml, 50 ng/ml, 100 ng/ml) according to ELISA results of gastric cancer cell lines were chosen to add to medium of preadipocyte differentiation-inducing mixture. The CCAAT enhancer binding protein alpha (C/EBPα), peroxisome proliferator activated receptor gamma (PPARγ) and delta like non-canonical Notch ligand 1 (DLK1) were key genes of regulating adipocyte differentiation. The protein encoded by Perilipin-1 coated lipid droplets which were positively correlated with adipocyte differentiation ratio and degree. The results showed that LAMC1 protein promoted preadipocyte differentiation, and the higher the concentration of LAMC1, the higher the triglyceride content in preadipocytes. Besides, lipid droplets were also seen outside the cells with the concentration of LAMC1 increasing in Oil Red O staining assay (Fig. 3A). Our results also confirmed that LAMC1 activated specific genes C/EBPα, PPARγ of adipogenesis and inhibited the production of DLK1 through the MEK/ERK signaling pathway, which caused more abundant lipid droplets accumulation in the preadipocyte in parallel with the upregulation of Perilipin-1 (Fig. 3A; Figure S2B). Different from preadipocyte, mature adipocyte had an ability to secrete adipokines, inflammatory cytokines, FFAs and lipid droplets. RT-qPCR results of adipokines and inflammatory cytokines showed that RNA levels were hardly affected under LAMC1 induction (Fig. 3B). RT-qPCR and western blots showed that 3T3-L1 had a higher HSL expression under LAMC1 stimulation (Fig. 3C). ELISA results showed that the CM (condition medium, CM) of 3T3-L1 under the induction of LAMC1 contains more free lipid droplets, FFAs, leptin and less adiponectin. The level of FFAs in 3T3-L1 CM was significantly higher than that of other adipokines (Fig. 3D). Moreover, FFAs in human preadipocytes CM were also elevated under LAMC1 induction (Fig. 3E). We also constructed an *in vitro* coculture model to simulate the interaction between tumor cells and preadipocytes in peritoneal microenvironment evaluating the effect of gastric cancer cell CM with LAMC1 knockout on preadipocytes differentiation (Fig. 3F). The results showed that the LAMC1 levels in LAMC1-knockout cancer cells CM were also decreased (Fig. 3G; Figure S2C). The 3T3-L1 cocultured with LAMC1 knockout CM had a lower C/EBPα, PPARγ, Perilipin-1 expression and higher DLK1 expression (Fig. 3H; Figure S2D). All results demonstrated that LAMC1 had the potential to accelerate preadipocytes differentiation and maturation. To further determine how adipocyte-derived adipokines, inflammatory cytokines, FFAs and lipid droplets functioned on gastric cancer cells, we used the adipocytes CM induced by LAMC1 to reverse co-culture with gastric cancer cell lines (Fig. 3I). Western blots showed that differentiated preadipocyte-derived CM promoted mesenchymal-epithelial transition in accordance with up-regulated E-cadherin and down-regulated vimentin (Fig. 3J). Furthermore, the results of CCK8 assay and Ki67 staining of cell slides revealed that LAMC1-induced CM enhanced the cell proliferation (Fig. 3K, Figure S3A-S3C). Preadipocytes were the dominant components of peritoneum, and we designed a coculture model *in vivo* to mimic preimplantation conditions of gastric cancer cells in peritoneal microenvironment, and our results showed that AGS/NC coculture group had a larger tumor size and weight (Fig. 3L). HE results demonstrated that the neoplastic cells from subcutaneous AGS/NC co-injection model showed more pleomorphic and hyperchromatic nuclei. Immunohistochemistry results showed that the LAMC1, E-cadherin, Perlipin-1 and Ki67 protein were upregulated, and vimentin was down-regulated in AGS/NC co-injection group (Fig. 3M). The above results reinforced that LAMC1 was a malignant tumor marker and LAMC1-mediated preadipocytes differentiation promoted peritoneum pre-metastatic niche formation. Mature adipocyte enhanced the ability of tumor proliferation and colonization.

### 3.4. Differentiated preadipocytes remodel metabolic programming of gastric cancer cells

**Figure 4:**
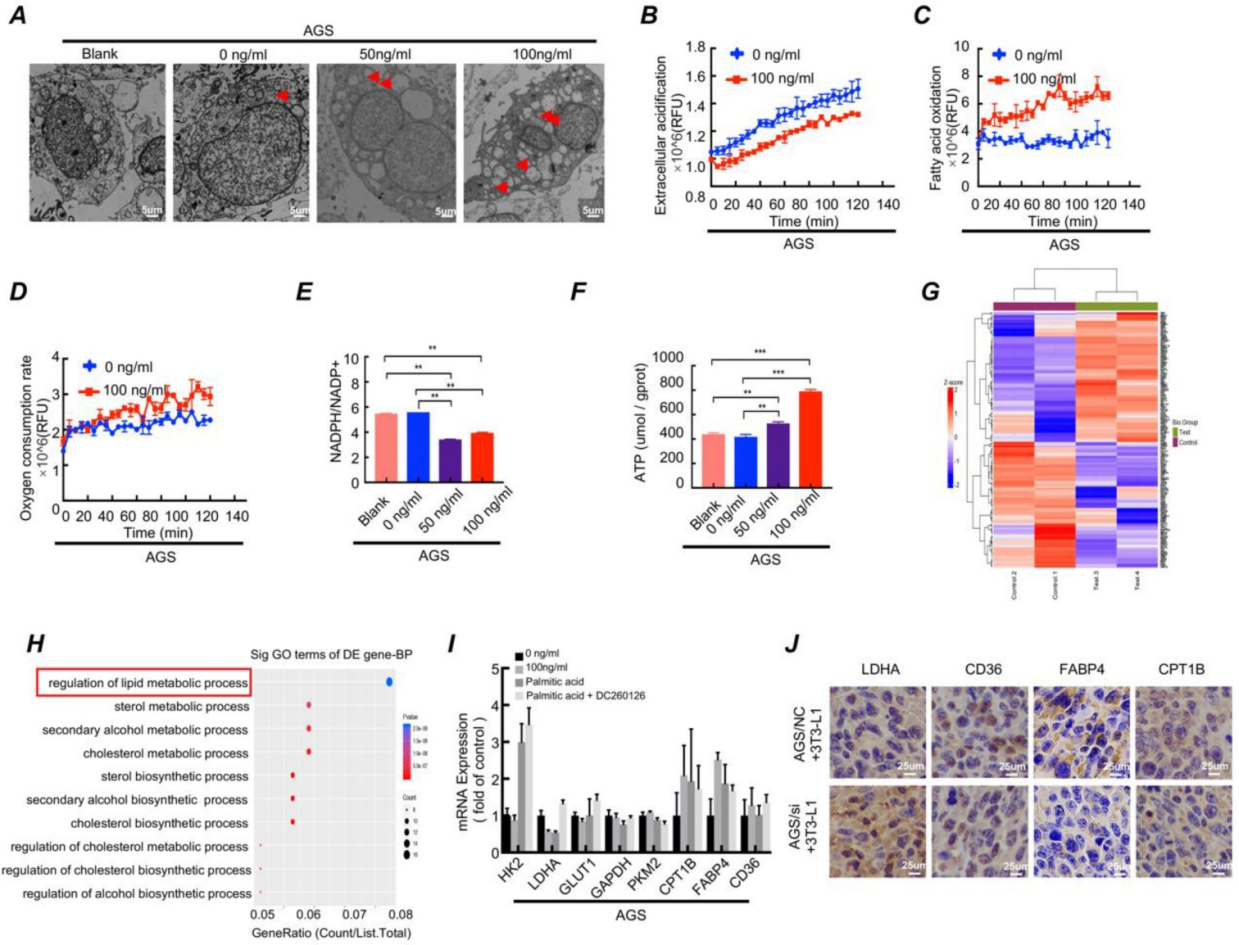
Differentiated preadipocytes remodel metabolic programming of gastric cancer cells. **(A)** Electron microscope was used to observe the content of lipid droplets in AGS cells after coculture for 48h with 3T3-L1 supernatant induced by LAMC1 (0, 50ng/ml and 100ng/ml) for 4 days. Blank group meant AGS cells weren't cocultured with 3T3-L1 supernatant.** (B-D)** The results of extracellular acidification, fatty acid oxidation and extracellular O_2_ consumption in AGS cells after reverse coculture for 48h was shown. **(E)** The ratio of NADPH / NADP+ in AGS cells after reverse coculture for 48h was assessed as materials and method.** (F)** The ATP content in AGS cells after reverse coculture for 48h was shown. **(G)** AGS cells treated with 0.5mM palmitic acid for 48h were analyzed by mass spectrometry. The Heat map of differential genes was shown.** (H)** AGS cells treated with 0.5mM palmitic acid for 48h were analyzed by mass spectrometry. The Gene function classification of differential genes was shown.** (I)** The RT- qPCR was for analyzing expression of metabolism-related genes in AGS cells after treatment with 0.5mM palmitic acid or reverse coculture with 3T3-L1 CM for 24h. 10uM DC260126 was used to inhibit the effect of palmitic acid. **(J)** The immunohistochemical results of xenograft tumor in AGS NC/si and 3T3-L1 coculture group. Error bars, SD. *P < 0.05; **P < 0.01; ***P < 0.001.

Differentiated preadipocytes was characterized by lipid droplets accumulation in the cytoplasm, and the secretion function of numerous lipid and protein factors^24^. Considering the characteristics of the differentiated preadipocyte, we conducted following series of experiments. The Oil Red O staining showed that lipid droplet content increased when gastric cancer cell cocultured with LAMC1-induced 3T3-L1 CM (Figure S3D). TEM results demonstrated that there was no presence of lipid droplets in the cytoplasm of gastric cancer cell lines that weren't co-cultured with adipocyte CM. Additionally, the higher the concentration of LAMC1 that induced 3T3-L1, the richer the content of lipid droplets in gastric cancer cells (Fig. 4A, Figure S3E). Glycolysis and β oxidation of fatty acids were key parts of metabolic programming, and our results showed that compared with 0 ng/ml LAMC1-induced 3T3-L1 CM, 100 ng/ml LAMC1 CM caused a reduced rate of extracellular acidification in gastric cancer and an increased rate of fatty acids oxidation (Fig. 4B-4C, Figure S3F-3G), moreover, oxygen consumption rates were also increasing in the 100 ng/ml LAMC1-induced 3T3-L1 CM coculture group (Fig. 4D, Figure S3H). NADPH was mainly derived from the pentose phosphate pathway and our results showed that the ratio of NADPH/NADP+ was declined in gastric cancer cell (Fig. 4E, Figure S3I). ATP was the most direct source of energy and gastric cancer cells produced more ATP after reverse co-culture (Fig. 4F, Figure S3J). All above results suggested that LAMC1-induced 3T3-L1 CM remodeled gastric cancer cell metabolic programming. FFAs as the most abundant component in supernatant might be involved in metabolic regulation. We chose the most abundant saturated fatty acids-palmitic acid to study effects of FFAs on tumor metabolism. AGS cells treated with 0.5mM palmitic acid for 48h were used for mass spectrometry analysis, and our results showed the different genes were involved in regulation of lipid metabolic process after palmitic acid stimulation (Fig. 4G-4H). RT-qPCR results showed that 100 ng/ml LAMC1-induced 3T3-L1 CM and palmitic acid inhibited key enzyme of glycolysis - LDHA expression and promoted fatty acid oxidation rate-limiting enzyme -CPT1B and FABP4 expression. DC260126- an effective FFAR1 (free fatty acid receptor 1, FFAR1) antagonist reversed the effect of palmitic acid (Fig. 4I, Figure S4A). We also verified the effects of preadipocytes on tumor metabolism *in vivo*, and immunohistochemistry results confirmed AGS/si group had a high-level LDHA expression. CD36, FABP4 and CPT1B were elevated slightly in AGS/NC coculture group (Fig. 4J). Our study showed LAMC1-mediated interaction between preadipocytes and gastric cancer cells remodeled tumor cell metabolic programming. Moreover, based on the results of co-culture between gastric cancer cells and preadipocytes, we considered that differentiated preadipocyte-derived palmitic acid played a vital role in metabolic regulation of gastric cancer peritoneal metastases, however, the molecular mechanism how palmitic acid affected gastric cancer remained unknown.

### 3.5. Palmitic acid phosphorylates STAT3 and promotes LAMC1 secretion through miR-193a-3p

**Figure 5:**
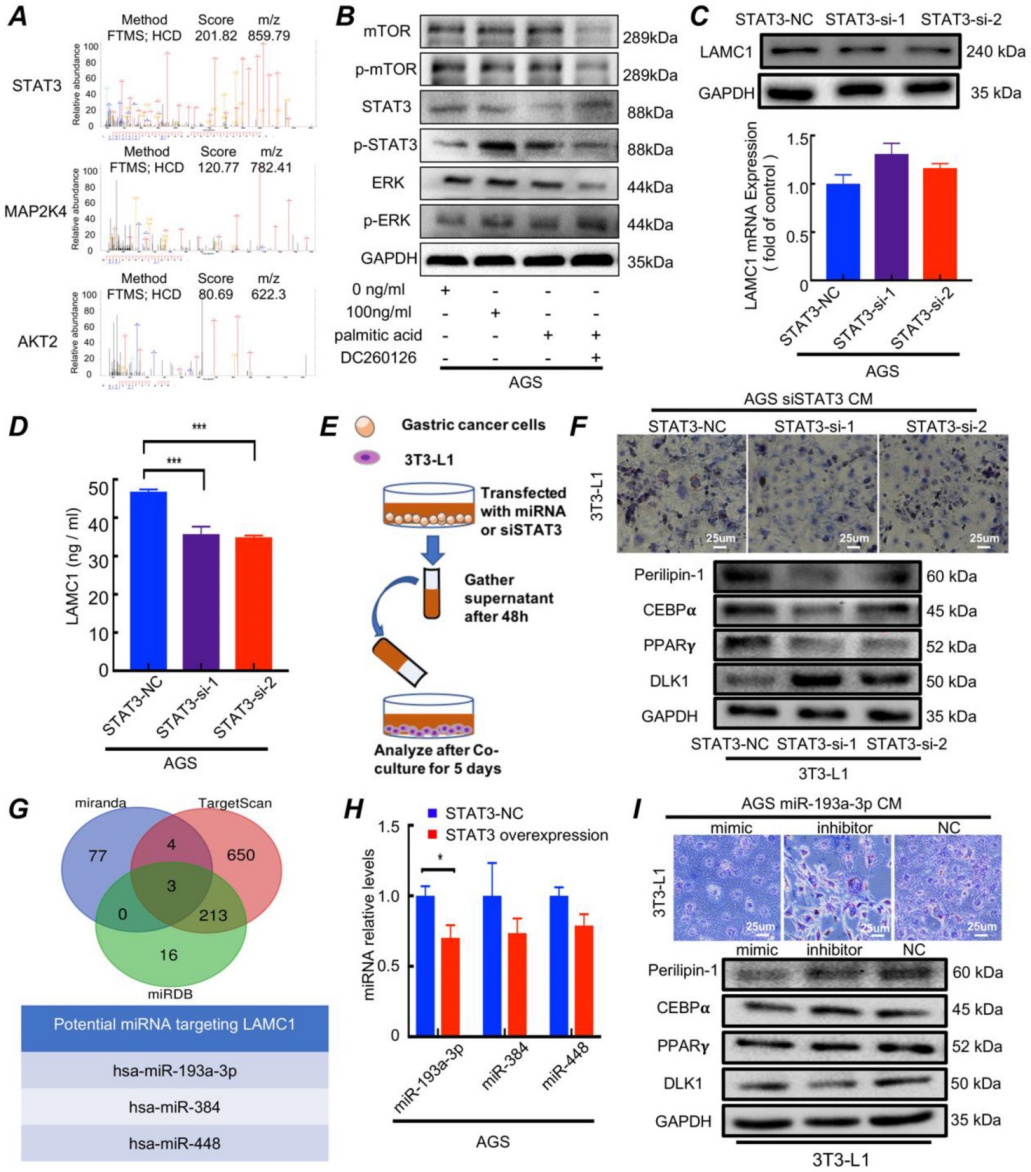
Palmitic acid phosphorylates STAT3 and promotes LAMC1 secretion through miR-193a-3p. **(A)** AGS cells treated with 0.5mM palmitic acid for 48h was used for mass spectrometry analysis and the three most abundant pathway proteins were shown. **(B)** The western blots were for analyzing pathway protein expression in AGS cells after treatment with 0.5mM palmitic acid for 48h or reverse coculture with 3T3-L1 supernatant induced by 0 and 100ng/ml LAMC1 for 48h. 10uM DC260126 was used to inhibit the effect of palmitic acid. **(C)** AGS cells transfected with siSTAT3 had a low LAMC1 expression determined by RT-qPCR, Western blots.** (D)** LAMC1 expression in supernatant of AGS cells transfected with siSTAT3.** (E)** Flowchart of co-culture of gastric cancer cell supernatant and 3T3-L1.** (F)** The gastric cancer cell supernatant transfected with siSTAT3 for 48h was collected to coculture with 3T3-L1 for 5 days. Western blots and the Oil Red O staining were used for lipid formation ability.** (G)** Three independent miRNA target databases were used to predict the potential miRNAs.** (H)** RT-qPCR was for analyzing miR193a-3p, miR384 and miR448 expression levels in AGS cells with STAT3 overexpression. **(I)** The gastric cancer cell supernatant transfected with miR-193a-3p mimic, inhibitor or NC for 48h was collected to coculture with 3T3-L1 for 5 days, and the Oil Red O staining and Western blots were used for lipid formation ability. Error bars, SD. *P < 0.05; **P < 0.01; ***P < 0.001.

Mass spectrometry results of AGS cells treated with 0.5mM palmitic acid for 48h showed MAP2K4, STAT3 and AKT2 were the three most abundant pathway proteins (Fig. 5A). There had been reports that palmitic acid could regulate MAPK/ERK^25^, STAT3^26^ and Akt/mTOR^27^ signaling pathway. Western blotting results showed that 100 ng/ml LAMC1-induced 3T3-L1 CM coculture group and palmitic acid activated STAT3, and when DC260126 inhibited the effect of palmitic acid, STAT3 had no activation. Moreover, palmitic acid had no effect on mTOR and ERK signaling pathway (Fig. 5B, Figure S4B). STAT3 as a core transcription factor might participate in LAMC1 expression. Our results demonstrated that gastric cancer cell with STAT3 knockout inhibited LAMC1 expression on protein levels significantly and STAT3 didn't affect LAMC1 mRNA expression (Fig. 5C, Figure S4C-4D). The ELISA results showed that LAMC1 in CM with STAT3 knockout was down-regulated (Fig. 5D, Figure S5A) and When STAT3-knockout gastric cancer cell CM to coculture with 3T3-L1 (Fig. 5E), the 3T3-L1 maintain the fibroblast phenotype in Oil Red O staining by decreasing the protein production of adipogenesis genes C/EBPα, PPARγ and Perilipin-1 and increasing DLK1 expression (Fig. 5F, Figure S5B), suggesting that STAT3 regulates the expression of LAMC1 in post-transcriptional level. Three independent databases TargetScan, mirnada and miRDB were used to predict miRNAs that targeted 3′ UTR of LAMC1 and hsa-miR-193a-3p, hsa-miR-384 and hsa-miR-448 were the candidate under taking intersection (Figure 5G). We further explored how STAT3 regulated miRNA and RT-qPCR results showed that STAT3 overexpression in AGS and BGC823 cell lines had a reduced miR-193a-3p expression, however, STAT3 had no influence on miR384 and miR-448 (Fig. 5H, Figure S5C). RT-qPCR results demonstrated that miR-193a-3p was downregulated in gastric cancer cell lines (Figure S5D). We transfected with gastric cancer cell lines with miR-193a-3p and transfected supernatant was collected to co-culture with 3T3-L1, the adipogenesis specific genes C/EBPα, PPARγ and Perilipin-1 were evidently elevated, and DLK1 was down-regulated in mimic group. The Oil Red O staining revealed that miR-193a-3p inhibited the formation of triglycerides (Fig. 5I; Figure S5E-5F). These results confirmed palmitic acid phosphorylated STAT3 and miR-193a-3p was a bridge between p-STAT3 and LAMC1.

### The molecular mechanism of the differentiation and maturation of 3T3-L1 cells

**Figure 6:**
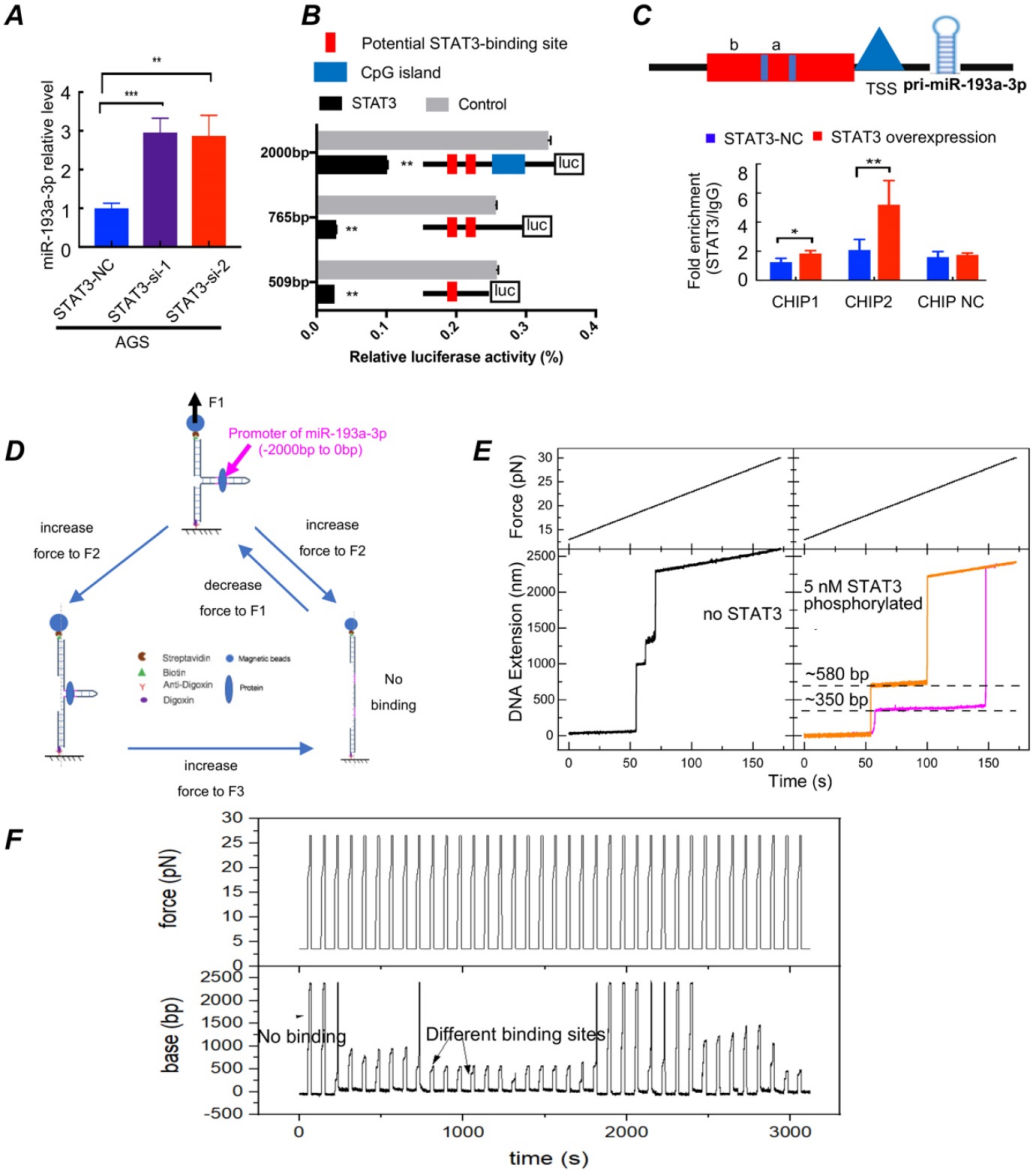
STAT3 activation downregulates miR-193a-3p expression. **(A)** AGS cells transfected with siSTAT3 had a high miR-193a-3p expression determined by RT-qPCR. **(B)** The PGL4 plasmids carrying different truncations of miR-193a-3p promoter and PCDNA3.1 vector containing STAT3 were transfected in 293T cells, and the relative luciferase activity was measured after 24h.** (C)** ChIP assay demonstrated the direct binding of STAT3 to the miR-193a-3p promoter.** (D)** Operation flow diagram of single-molecular magnetic tweezers. **(E-F)** Force vs. time and DNA extension vs. time curve for promoter of miR-193a-3p with or without p-STAT3. Error bars, SD. *P < 0.05; **P < 0.01; ***P < 0.001.

**STAT3 activation downregulates miR-193a-3p expression (Fig. 6)**. To clarify whether palmitic acid upregulated the expression of LAMC1 through inhibition of miR-193a-3p or not, we silenced the expression of STAT3. RT-qPCR results showed different STAT3 knockout group recovered the transcription level of miR-193a-3p in AGS and BGC823 cell lines (Fig. 6A, Figure S6A). The results of the STAT3 expression in two transfection groups revealed that LAMC1 and miR-193a-3p couldn't regulate STAT3 (Figure S6B-6C). Above results showed that STAT3 regulated LAMC1 through miR-193a-3p in a post-transcription and one-way manner. According to bioinformatics analysis, we found that promoter region between -2000bp and -780bp of miR-193a-3p had a large number of methylation sites, and -770bp/0 consisted of potential binding sites of STAT3 in miR-193a-3p promoter region (Figure S6D-6E). In order to determine whether STAT3 worked by methylation or directly bound to the promoter region, we conducted the following experiments. A luciferase reporting experiment by constructing the full length -2000bp/0 of promoter revealed that STAT3 transcriptionally suppressed miR-193a-3p and methylase inhibitor zebularine didn't change the negative regulation of STAT3 on miR-193a-3p (Figure S7A). In order to further exclude the influence of other methylation sites and clarify the specific binding sites of STAT3 in the miR-193a-3p promoter region, its truncation (-509/0, -765/0) were constructed based on Jaspar score, and luciferase reporting experiments found that two candidate fragments were responsible for STAT3-mediated promoter regulation (Fig. 6B). To further explore binding mechanism, we designed two pair primers to amplify promoter regions of miR-193a-3p predicted in Jaspar containing two candidate binding sites and found that 174bp (-509bp ~ -335bp) and 256bp (-765bp ~ -509bp) were responsible for STAT3 regulating (Fig. 6C). A high-resolution magnetic tweezer was used in our results to explore binding mechanics between STAT3 and promoter region at single-molecule levels and we found that phosphorylation had an important influence on DNA binding of STAT3, furthermore, STAT3 didn't bind to promoter of miR-193a-3p without ATP which was consistent with our *in vitro* cell experiments that crosstalk between adipocytes and gastric cancer cells provided amounts of ATP for tumor cells. Its potential binding sites located near 350bp and 580bp. Actived-STAT3 protein formed homo- or hetero-dimers^28^ and our results showed that only when the dimer structure, the binding sites and ATP existed simultaneously, p-STAT3 bound to the promoter of miR-193a-3p smoothly. The combining structure was very stable and withstood a pulling force greater than 22pN. When the opening double-stranded DNA restored base pairing, the protein combined with DNA quickly (Fig. 6D-6F; Figure S7B). All results confirmed that STAT3 has multi-site regulation on the miR-193a-3p promoter region and it formed a stable binding in a few seconds and inhibit the expression of miR-193a-3p.

**Figure 7:**
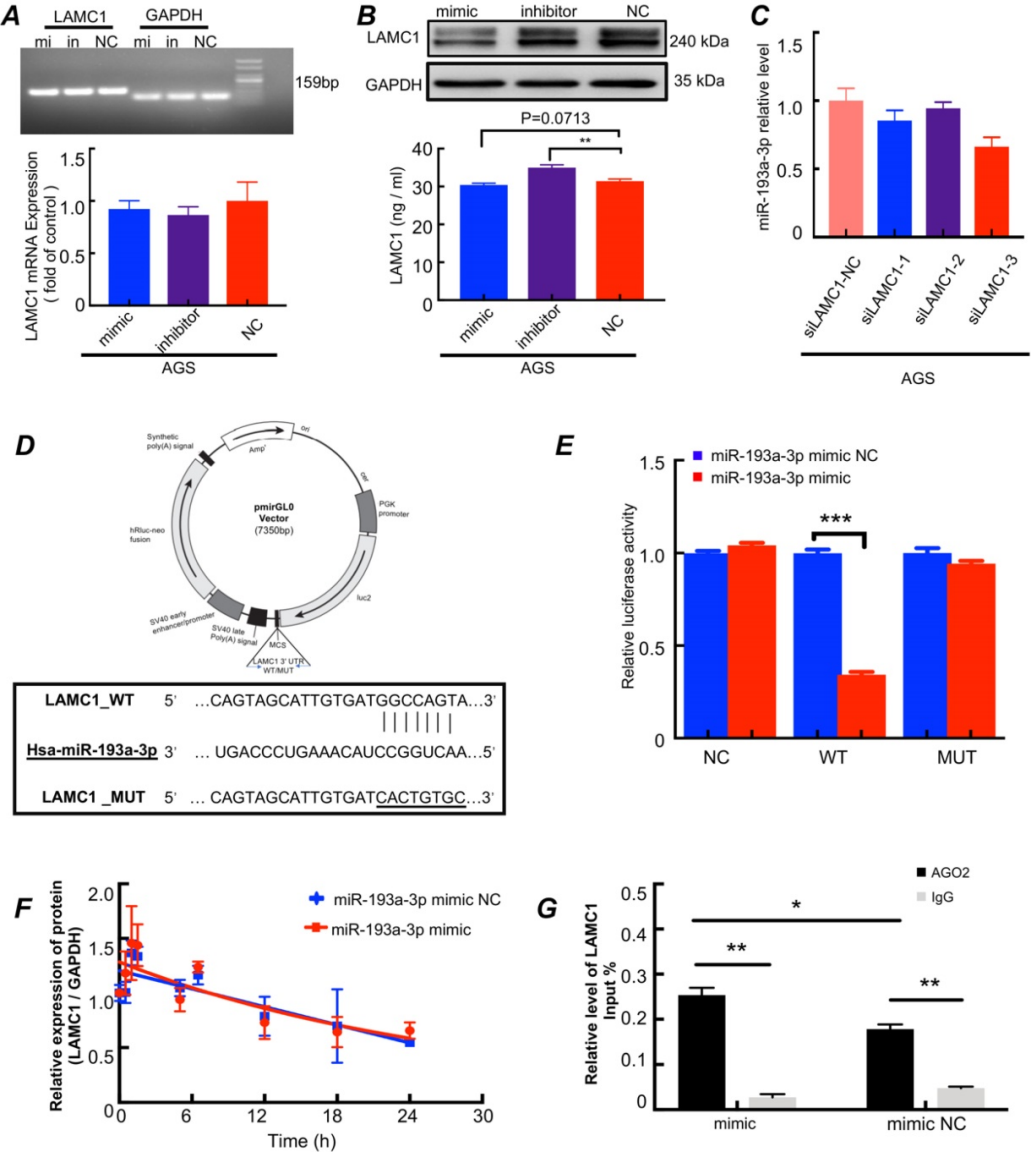
The miR-193a-3p inhibits LAMC1 expression in a post-transcriptional manner. **(A-B)** AGS cells were transfected with miR-193a-3p mimic, inhibitor or NC, and agarose gel electrophoresis and RT-qPCR were used to measure RNA level change, Western blots and ELISA for protein level. **(C)** The AGS cells were transfected with siLAMC1, RT-qPCR for miR-193a-3p expression. **(D)** A structure diagram of the pmirGLO dual-luciferase reporter vector with 3′-UTR of LAMC1 mRNA harbors miR-193a-3p binding sites. **(E)** The relative luciferase activity of reporter plasmids carrying wild-type or mutant LAMC1 3′-UTR. **(F)** The LAMC1 mRNA decay curves of AGS cells carrying miR-193a-3p mimic or mimic NC. **(G)** The AGS cells were transfected with miR-193a-3p mimic or mimic NC for 48h, and AGO2-RNA immunoprecipitation assay (RIP) was used for analyzed the combination method of miR-193a-3p and LAMC1. Error bars, SD. *P < 0.05; **P < 0.01; ***P < 0.001.

**The miR-193a-3p inhibits LAMC1 expression in a post-transcriptional manner (Fig. 7).** To confirm whether LAMC1 was a direct target of miR-193a-3p, we conducted a series of experiments and found that after transferred with miR-193a-3p mimics, miR-193a-3p inhibitor or negative control, the RNA levels of LAMC1 were not changed. And the agarose gel electrophoresis experiment confirmed the specificity of the primers and further explained that the RNA content of LAMC1 was basically the same by comparing the brightness of the bands under ultraviolet light (Fig. 7A, Figure S7C). At the protein level, LAMC1 was obviously down-regulated after transfection with mimic, but the inhibitor group wasn't much different from the NC group, which was caused by the ineffective inhibitory effect of the transfection reagent, and comprehensive ELISA results showed that after miR-193a-3p transcription was inhibited, LAMC1 expression was augmented, and the supernatant content of LAMC1 was also increased, and vice versa (Fig. 7B, Figure S7D). When the intracellular LAMC1 gene was knocked out, the transcription of miR-193a-3p was completely unaffected (Fig.7C, Figure S7E). The study showed from the reverse side that miR-193a-3p regulated LAMC1, but the presence or absence of LAMC1 had nothing to do with miR-193a-3p indicting miR-193a-3p was the messenger of IL6 and LAMC1. We used a 3′-UTR of luciferase reporter plasmids containing miR-193a-3p binding sequences to analyze the mutual relationship (Fig. 7D). Compared with the mutations, the overexpression of miR-193a-3p abolished the luciferase activities of the LAMC1 3′-UTR reporter constructs (Fig. 7E). To further analyzed the specific role of miR-193a-3p for LAMC1 expression, actinomycin D was added to AGS cell line and LAMC1 mRNA levels were detected at designated time points. LAMC1 decay curves of AGS cells normalized by GAPDH carrying miR-193a-3p mimic or mimic NC revealed that miR-193a-3p didn't influence LAMC1 mRNA stability (Fig. 7F). Then AGO2-RNA immunoprecipitation assay (RIP) was performed, and the results showed that LAMC1 mRNA was enriched in the AGO2 immunoprecipitates compared with IgG. Furthermore, the mimic group was higher than the NC group in terms of LAMC1 mRNA enriched by AGO2 (Fig. 7G). Considering the results, STAT3 directly inhibited miR-193a-3p transcription, and miR-193a-3p repressed LAMC1 translation through post-transcriptional regulation. Collectively, our study deciphered the molecular mechanisms that p-STAT3 / miR-193a-3p / LAMC1 axis was an essential signaling pathway of gastric cancer peritoneal metastasis.

### 3.7. Gastric cancer with high LAMC1 expression has high risk of peritoneal metastasis

**Figure 8:**
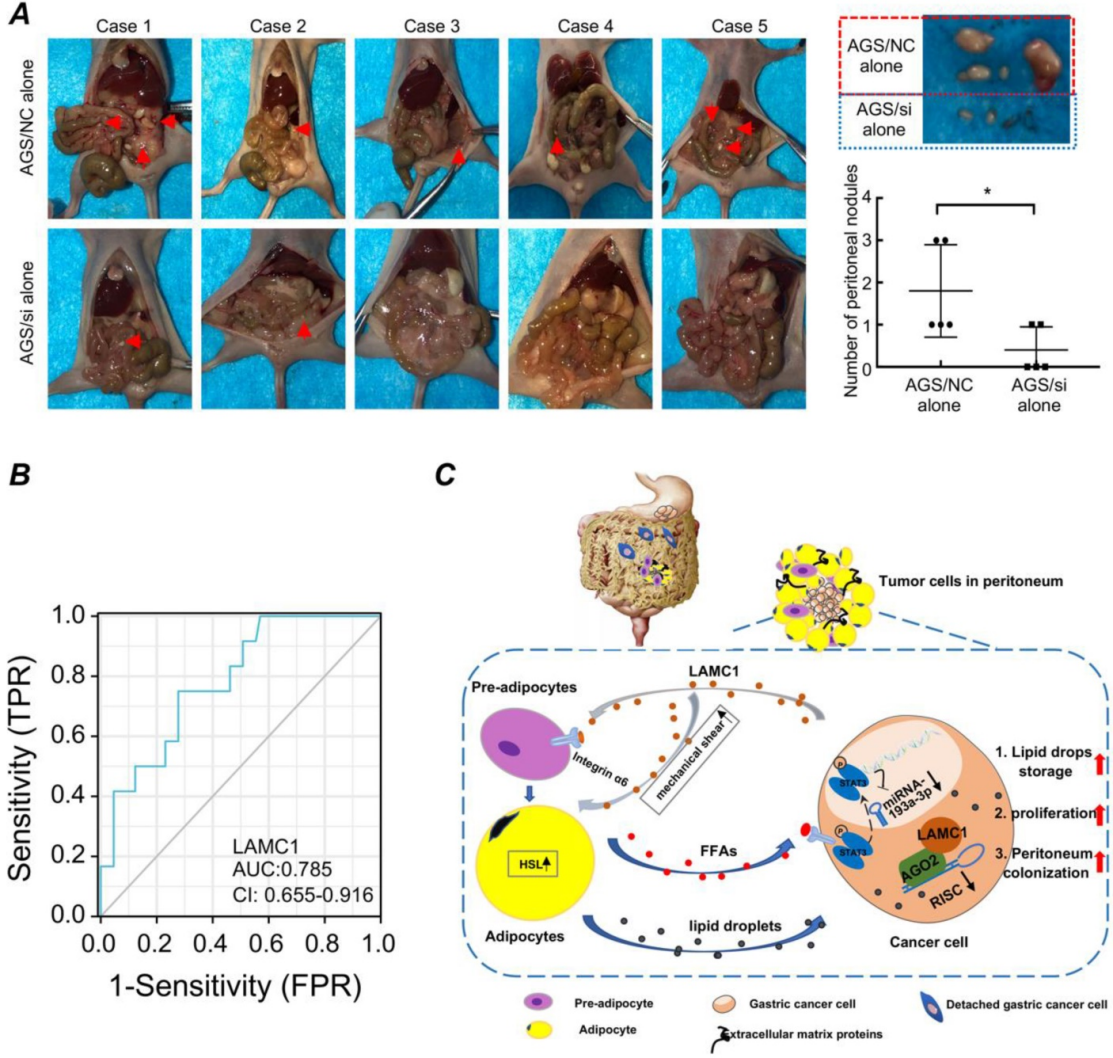
Gastric cancer with high LAMC1 expression has high risk of peritoneal metastasis. **(A)** Gross anatomy and nodule numbers of abdominal cavity in nude mice and the red arrows indicated micrometastases in the gastric cancer peritoneal metastasis model. **(B)** ROC curve.** (C)** The mechanism diagram of LAMC1-mediated preadipocytes differentiation promoted peritoneum pre-metastatic niche formation and gastric cancer metastasis.

Based on the above experimental results, we designed *in vivo* animal model to further explore the relationship between LAMC1 and peritoneal metastasis. Our results showed that when the tumor was injected intraperitoneally to simulate the preimplantation state of shedding tumor cells, AGS/NC groups had a multifocal peritoneal metastasis, especially in adipose tissue-rich omentum, moreover, the tumor size of AGS/NC groups were larger than AGS/si groups (Fig. 8A). We also collected blood from 77 patients, and according to the presence or absence of peritoneal metastasis, we divided all patients into two groups. ELISA was used to detect the LAMC1 expression and SPSS was used to plot ROC curve. Our results showed that high LAMC1 expression in serum indicated a high risk of peritoneal metastasis, and the area under the curve was 0.785 (Fig. 8B). Above results demonstrated that gastric cancer with high LAMC1 expression had high risk of peritoneal metastasis.

## Discussion

In our study, we found that gastric cancer cells secreted LAMC1 to obtain mobility ability by autocrine mode and promote preadipocyte maturation in a paracrine manner. Mature adipocytes secreted abundant FFAs to remodel gastric cancer cell metabolic programming by inhibiting glycolysis and promoting oxidation of fatty acids. Moreover, differentiated preadipocyte-derived CM enhanced the ability of proliferation and mesenchymal-epithelial transformation of gastric cancer cell which facilitated peritoneal implantation of gastric cancer. FFAs-activated p-STAT3/miR-193a-3p/LAMC1 axis played an important feedback regulation role in the reciprocal interaction between gastric cancer cells and preadipocytes which directly contributed to gastric cancer peritoneal metastasis (Fig. 8C).

The onset of adipogenesis is defined by extracellular matrix remodeling, and many extracellular matrix proteins can promote adipocyte differentiation. In our study, the high concentration of LAMC1 in the medium is conducive to the accumulation of lipid droplets of adipocytes, so we surmised that extracellular matrix beneficial to adipocyte differentiation is characterized by excessive laminin. The research by Ferdinand P. Brandl reported that preadipocytes tended to form a polygonal shape in the culture plate which wasn't conducive to the accumulation of lipid droplets, but 3T3-L1 cells in enzymatically degradable poly (ethylene glycol) based hydrogels was rounded and had a high adipocyte differentiation proportion^29^. Laminin encoded by LAMC1 is more inclined to assemble a loose three-dimensional structure and preadipocytes in that structure are prone to appear as rounded morphology derived from cytoskeleton remodeling through MEK/ERK signaling pathway to accelerate visual lipid droplets growth. On the other hand, the overexpression of LAMC1 improves the matrix rigidity and forms a milieu that exerts more contractile force for adipocyte rupture which is consistent with our results that when human recombinant LAMC1 was added to the medium, lipid droplets appeared in the supernatant. When the lipid-rich CM was co-cultured with gastric cancer cells, lipid droplets were also seen in the gastric cancer cells. SPARC a gene that encoded cysteine-rich acidic matrix-associated protein could suppress adipogenesis and decrease homing of ovarian cancer cells to omental adipocytes via inhibition of cEBPβ-NFkB-AP-1 transcription machinery^30^. The protein encoded by THBS1 is an adhesive glycoprotein that can bind to fibrinogen, fibronectin, laminin, type V collagen and integrins to participate in extracellular matrix remodeling. THBS1 can facilitated fatty acid uptake and proliferation of adipocyte^31^ and the results of obesity hypoventilation syndrome study elaborated that log-term HFD feeding could active THBS1 to enhance intra-diaphragmatic fibro-adipogenic progenitors (FAPs) proliferation giving rise to more adipocytes and fibroblasts^32^. Matricellular protein CTGF containing a novel fibronectin binding site enhanced the capability of cell adhesion and migration^33^. The overexpression of CTGF inhibited 3T3-L1 preadipocytes to differentiate into mature adipocytes^34^. COL1A1^35^ and Fn1^36^ were also restricted to preadipocyte.

Moreover, we also found the reduced ratio of NADPH/NADP+ and increased ratio of ATP in gastric cancer cells. NADPH was mainly derived from the pentose phosphate pathway^37^ and the results showed that 100 ng/ml LAMC1-induced 3T3-L1 CM inhibited glucose metabolic pathway. Subsequent experimental results also confirmed that adipocyte-derived CM inhibited glycolysis and promoted oxidation of fatty acids which provided ATP for tumor growth and metastasis. High concentration of ATP could inhibit aerobic glycolysis known as the Warburg effect, and ATP consumption caused a compensatory increase which could maintain glycolytic flux^38^. NADPH played an important role in redox regulation through regeneration of glutathione (GSH) to combat reactive oxygen species (ROS) meeting their own survival needs 39. The cellular levels of NADPH were decreasing during lipid droplet synthesis and increasing during fatty-acid oxidation. Large amount of ROS could increase the probability of pro-oncogenic mutations and the research by Esther Aguilar^40^ pointed out that increased ROS production due to NADPH pool deficiency was beneficial for initiation of tumorigenesis and malignant transformation.

Tumor metastasis is a complex process in which tumor cells continuously adapt and reprogram the microenvironment. Our research demonstrated that the elevated LAMC1 expression was related to poor prognosis of patients with gastric cancer which was consistent with many studies that LAMC1 promoted malignant tumor progression^41^-^43^. Namani A et al.^44^ showed that high expression of LAMC1 regulated by Nuclear factor erythroid-derived-2-like 2 (NRF2) promoted migration and invasion of A549 non- small cell lung cancer cells. Moreover, Yuhui Yu et al.^45^ also reported that LAMC1 knockout inhibited tumor growth and malignant phenotypes development in lung cancer. The LAMC1 protein contains 1609 amino acids in UniProt database. Due to technical limitations, we couldn't construct the LAMC1 overexpression plasmid, so it was our limitations that only LAMC1 knockout cell lines were used to compare and judge the change of invasion and migration ability of gastric cancer cells. In our study, when LAMC1 wasn't knockdown in gastric cancer and LAMC1 expression was at a high level, the migration and invasion ability of gastric cancer was significantly upregulated at the primary site. Furthermore, differentiated preadipocyte-derived supernatant promoted tumor mesenchymal-epithelial transformation (MET). Nikitha K. Pallegar's study^9^ showed that in 3D coculture model, adipocytes and adipocyte-derived conditioned media, not preadipocytes caused the breast cancer cell to form epithelial morphology, however, highly diluted condition medium lost the function of induction. In our study, tumor cells in peritoneal microenvironment were surrounded by adipose tissue and MET occurred in tumor cells.

At present, with regard to the mechanism by which adipocytes promote cancer peritoneal metastasis, the most studied theories include the following four: (1) Adipocytes up-regulate CD36^46^, fatty acid binding protein 4 (FABP4)^47^ and Phosphatidylinositol transfer protein, cytoplasmic 1 (PITPNC1) expression^48^ to accelerate fatty acids transport and oxidation in order to satisfy own energy requirement. (2) Adipocytes can control macrophage phenotype changes by recruiting macrophages to increase the proportion of M2 macrophages^49^. (3) Adipocytes secrete exosomes to promote tumor distant metastasis^50^, ^51^. (4) Adipocytes secrete a wealth of adipokines, such as IL-6 and leptin, activating the intracellular signaling pathway to increase the malignancy of the tumor^52^. As for adipokines, Uddin S and his colleagues^53^ proved that the obesity hormone leptin combined to its receptor Ob-R to produce biological activity through the PI3K signaling pathway, leading to a significant poor disease-free survival for patients with ovarian cancer. In human chondrosarcoma cells, resistin induced angiogenesis of endothelial progenitor cells via upregulating VEGF-A expression through PI3K/Akt/miR-16-5P/VEGF-A signaling pathway to promote chondrosarcoma metastasis^54^. Nakajima TE and his colleagues^55^ used a case-control study to demonstrate that blood concentrations of adipokines were related to the risk of gastric cancer and the biomarkers of the progression of gastric cancer included leptin, resistin, visfatin and C-peptide. However, how adipocyte-derived FFAs affecting LAMC1 and metabolic regulation promotes gastric cancer progression is still unclear.

In our study, palmitic acid - a type of saturated fatty acid, inhibited glycolysis and promoted oxidation of fatty acids in gastric cancer cells. Other study pointed out that polyunsaturated fatty acid, especially arachidonic acid (AA) and linoleic acid (LA), had a good predictive value for the identification of lung adenocarcinoma^56^. Romana T.Netea-Maier et al.^57^ identified that high concentration of AA and LA in tumor ascites could shift glucose catabolism of tumor-associated macrophages (TAMs) to aerobic glycolysis which was beneficial for tumor to adapt to hypoxic environment. Meanwhile, palmitic acid could induce endoplasmic reticulum stress and promote tumor metastasis^58^, however, Lin's team^59^ had an opposite observation that palmitic acid impaired hepatocellular carcinoma development by inhibiting mTOR and STAT3 pathway activation which also contradicted our results that adipocyte-derived palmitic acid activated STAT3 in gastric cancer. However, palmitoylated STAT3 by palmitic acid was more inclined to membrane recruitment and phosphorylation by JAK2 in inflammatory bowel disease^26^. The above results led us to surmise that palmitic acid had a different effect on STAT3 in different disease.

Moreover, we are the first to demonstrate a positive correlation between palmitic acid and the expression of LAMC1. Our results also disclose that there is a negative correlation between palmitic acid and LAMC1 with miR-193a-3p in gastric cancer cells and LAMC1 is the target of miR-193a-3p regulated by p-STAT3. MiRNAs are short non-coding RNAs involved in key biological processes, which can degrade mRNA or perform post-transcriptional regulation^60^. In cancer, many miRNAs are suppressed because they are located in fragile sites in the genome, and their high expression can inhibit the invasion and metastasis of cancer, therefore, miRNA-based therapies show great potential in various tumors^61^. MiR-193a-3p is a powerful tumor suppressor gene, participating in non-small cell lung cancer^62^, colorectal cancer^63^ and breast cancer^64^ progression. Chen K et al^65^ found that miR-193a-3p methylation silencing could target up-regulation of related genes GRB7, ERBB4, SOS2 and KRAS in the downstream of MAPK / ERK signaling cascades, resulting in the potent modulation of ovarian cancer invasion and metastasis, and its inhibitory effect on KRAS also had a potential role in reducing circulating tumor cells in the blood^66^. STAT3-miRNAs interactions are emerging as a novel mechanism of malignant tumor regulation reacted in many literatures. Guang-Yuh Chiou and his co-workers found that IL6 could induce DNA methyltransferase 1 expression leading to hypermethylation of the miR142-3p promoter which influenced Sp1-binding motif in the promoter to contribution to glioblastoma tumorigenesis^67^. Hypoxia-induced protective autophagy via activation of p-STAT3-miR-155-3p-CREBRF-CREB3-ATG5 pathway could accelerate malignant glioma progression^68^. A TrkB-STAT3-miR-204-5p regulatory circuitry was corroborated to play a critical role in controlling proliferation and invasion of endometrial carcinoma cells^69^. The STAT3-miRNA-92-Wnt signaling pathway was beneficial for ovarian cancer spheroids formation and generation of cancer stem-like cells^70^. In our study, p-STAT3/miR-193a-3p/LAMC1 signaling axis is corroborated to have a powerful effect in metastasis of gastric cancer to adipocyte-rich peritoneum which provides a new sight for targeted therapy.

## Conclusion

In summary, our study disclosed that the augmented expression of LAMC1 was related to preadipocyte differentiation and pre-metastatic niche formation. The differentiated preadipocytes served as energy storage reservoir and secreted FFAs to remodel tumor metabolic programming, moreover, differentiated preadipocyte-derived CM was beneficial for proliferation and colonization. Totally, palmitic acid/p-STAT3/miR-193a-3p/LAMC1 regulation circuitry was favorable for gastric cancer peritoneal metastasis. LAMC1 was a prediction marker gene for gastric cancer peritoneal metastasis and it might be a potential therapeutic target for gastric cancer progression.

## Supplementary Material

Supplementary figures and tables.

## Figures and Tables

**Table 1:** Correlation between the density of LAMC1 at gastric cancer and clinicopathologic parameters

Parameters	N (%)	LAMC1 expression		
		High	Low	P
Gender				0.0628
Male	64 (71.11)	28	36	
Female	26 (28.29)	17	9	
Age (years)				0.3894
<60	36 (40.00)	16	20	
≥60	54 (60.00)	29	25	
T stage				**0.0112**
T1-2	20 (22.22)	5	15	
T3-4	70 (77.78)	40	30	
N stage				0.6726
N0-1	48 (53.33)	23	25	
N2-3	42 (46.67)	22	20	
TNM stage				
I/II	35 (38.89)	12	23	**0.0174**
III	55 (61.11)	33	22	
CEA (ng/ml)				0.3574
<7.2	85 (94.44)	41	44	
≥7.2	5 (5.56)	4	1	
CA125 (U/ml)				>0.9999
<35	83 (92.22)	41	42	
≥35	7 (7.78)	4	3	
CA199 (U/ml)				0.3343
<37	79 (87.78)	38	41	
≥37	11 (12.22)	7	4	
Peritoneal metastasis				**0.0243**
YES	11 (12.22)	9	2	
NO	79 (87.78)	36	43	

## Abbreviations

C/EBPα: CCAAT enhancer binding protein alpha
DLK1: delta like non-canonical Notch ligand 1
FAO: fatty acid oxidation
FFAs: free fatty acids
LAMC1: laminin gamma 1
PPARγ: peroxisome proliferator activated receptor gamma
