# Supplementary material for: LAMC1-mediated preadipocytes differentiation promoted peritoneum pre-metastatic niche formation and gastric cancer metastasis
Source: Int J Biol Sci. 2022 Apr 24;18(7):3082–101. doi: 10.7150/ijbs.70524 (PMC9066104; doi:10.7150/ijbs.70524)
Supplement: Supplementary file 1 — Supplementary figures and tables. [file ijbsv18p3082s1.pdf]

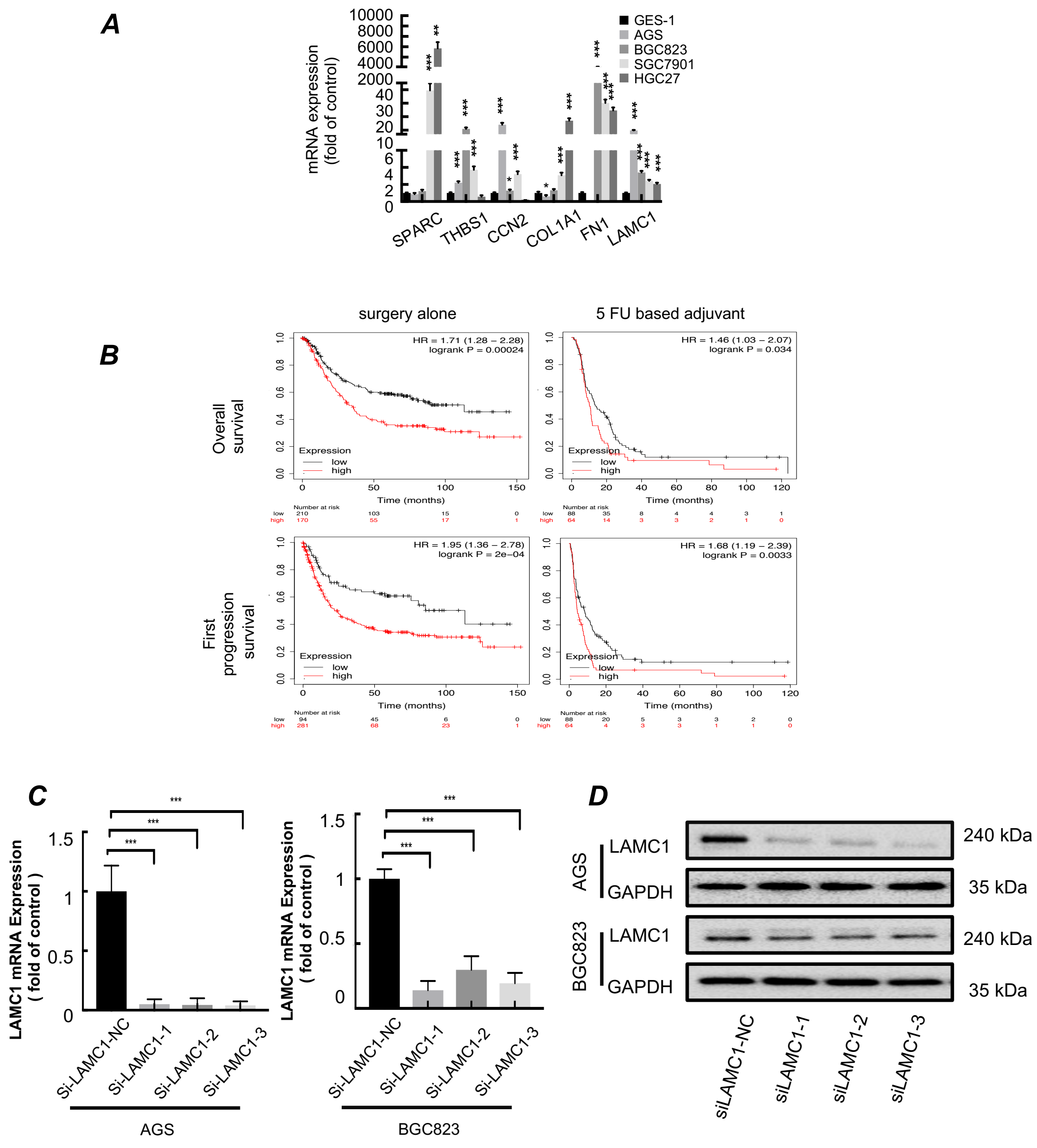

Supplementary Fig. 1 (A) RT-qPCR for analyzing differential expression of extracellular matrix proteins in gastric cancer cell lines. **(B)** Effect of LAMC1 expression level on overall survival and first progression survival of gastric cancer patient with Kaplan-Meier plotter. **(C-D)** The RT- qPCR and Western blots for analyzing transfection efficiency in gastric cancer cell lines transfected with siLAMC1. Error bars, SD. *P < 0.05; **P < 0.01; ***P < 0.001.

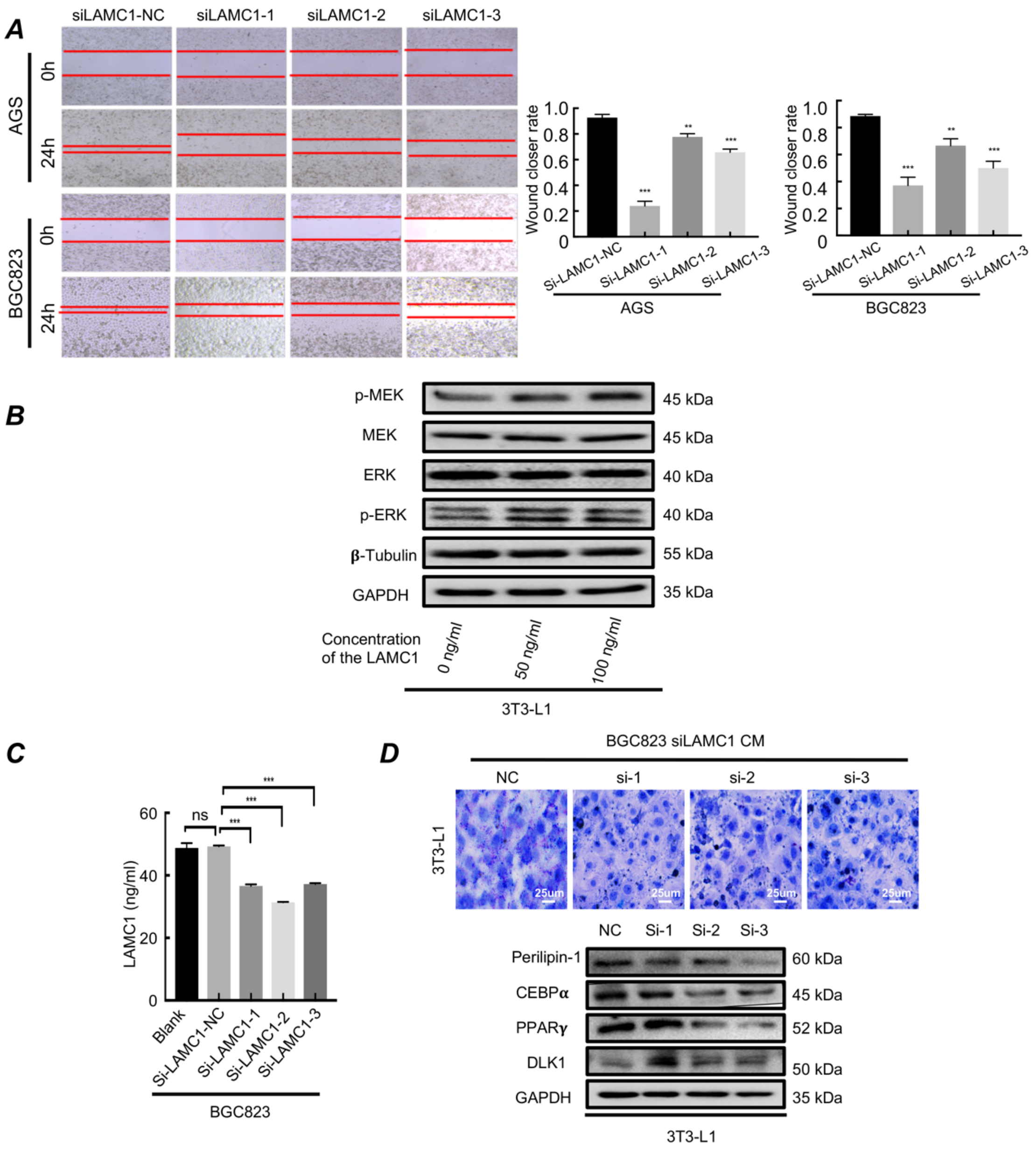

Supplementary Fig. 2 (A) Cell scratch test in gastric cancer cells with LAMC1 knockout. **(B)** The effect of the LAMC1 on downstream pathway protein was analyzed in 3T3-L1 cells by Western blots. **(C)** The ELISA results for analyzing transfection efficiency in gastric cancer cell lines transfected with siLAMC1. **(D)** The lipid droplet formation ability of 3T3-L1 was measured by Oil Red O staining and adipocyte differentiation related protein after coculture for 5 days with gastric cancer cell supernatant transfected siLAMC1 for 48h. Error bars, SD. *P < 0.05; **P<0.01; ***P < 0.001.

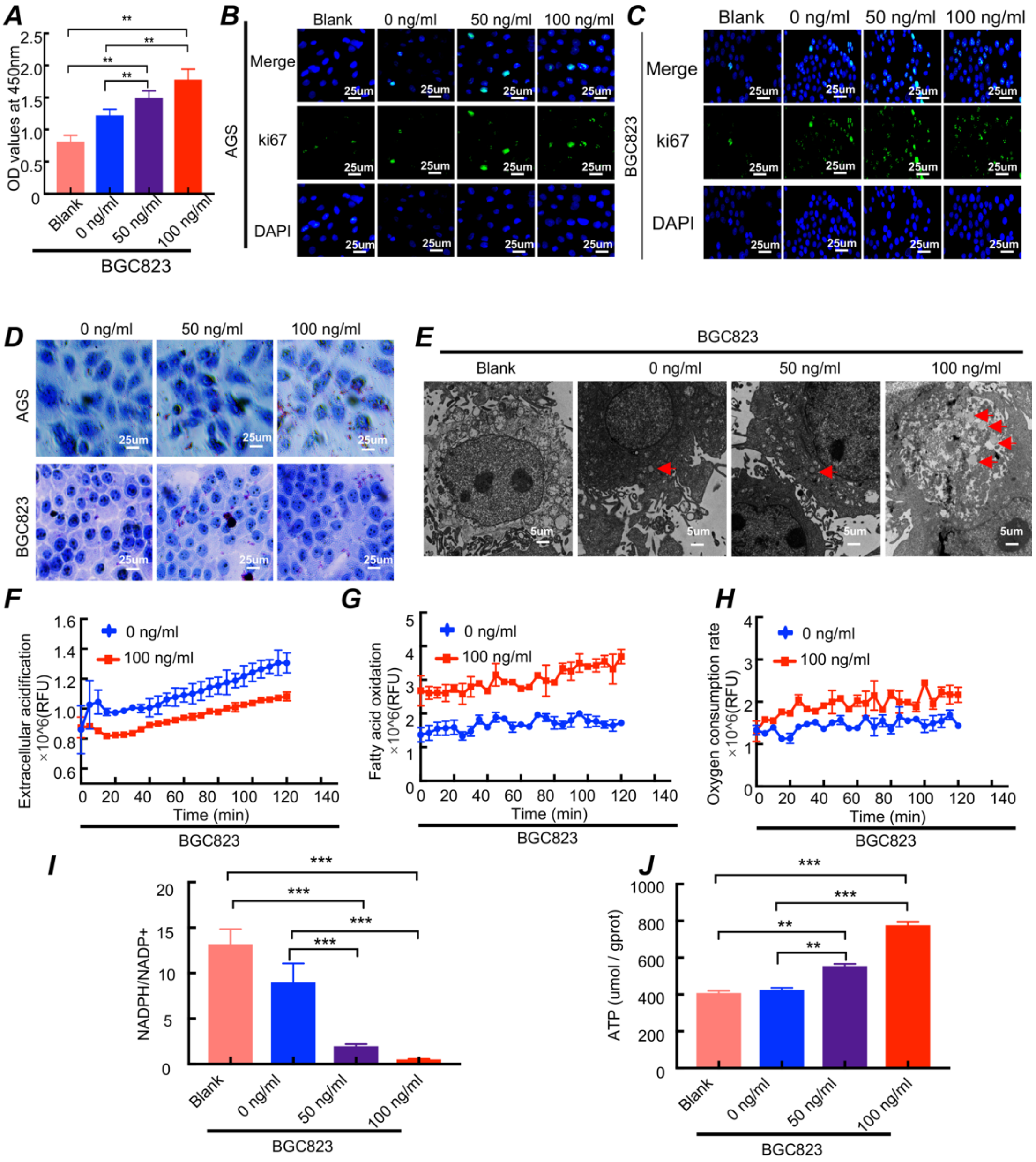

Supplementary Fig. 3 (A) CCK8 assay for cell proliferation detection**. (B-C)** The cell slide immunofluorescence staining of ki67. **(D-E)** The supernatant of 3T3-L1 induced with LAMC1 (0, 50ng/ml and 100ng/ml) was collected to coculture with AGS and BGC823 cells. The Oil Red O staining and Electron microscope were for analyzing lipid uptake of gastric cancer cells. Blank group meant AGS cells weren't cocultured with 3T3-L1 supernatant. **(F-H)** The analysis of extracellular acidification, fatty acid oxidation and extracellular O_2 consumption in BGC823 cells after reverse coculture for 48h with 3T3-L1 supernatant induced by LAMC1 for 4 days was assessed as materials and method. **(I)** The ratio of NADPH/NADP+ in BGC823 cells after reverse coculture for 48h with 3T3- L1 supernatant induced by LAMC1 for 4 days was assessed as materials and method. **(J)** The ATP content in BGC823 cells after reverse coculture for 48h with 3T3-L1 supernatant induced by LAMC1 for 4 days was assessed as materials and method. Error bars, SD. $^{*}P < 0.05$; $^{**}P < 0.01$; $^{***}P < 0.001$.

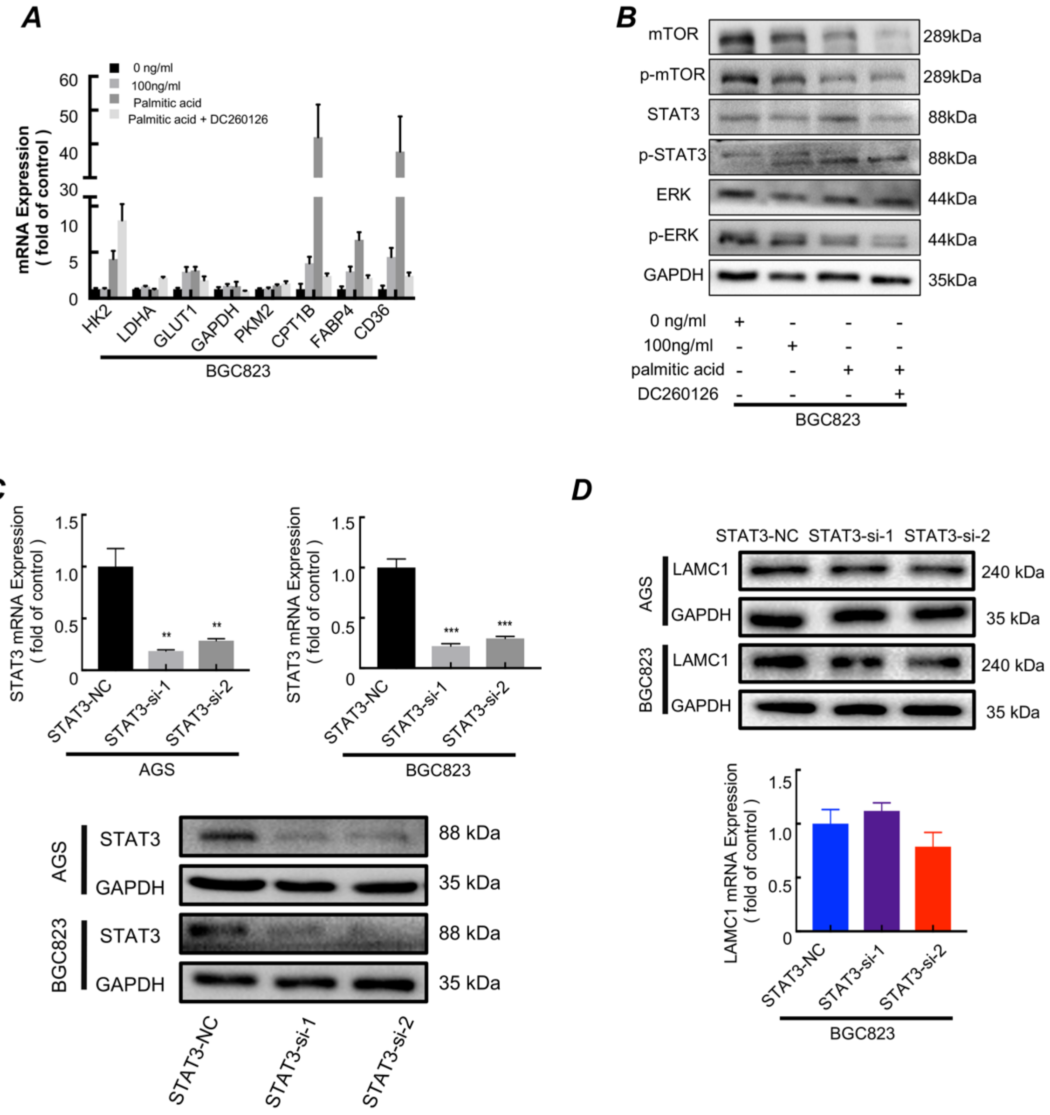

Supplementary Fig. 4 (A) The RT- qPCR was for analyzing expression of metabolism-related genes in BGC823 cells after treatment with 0.5mM palmitic acid for 24h or reverse coculture with 3T3-L1 supernatant induced by 0 and 100ng/ml LAMC1 for 48h. 10uM DC260126 was used to inhibit the effect of palmitic acid. **(B)** The Western blotting was for analyzing pathway protein expression in BGC823 cells after treatment with 0.5mM palmitic acid for 48h or reverse coculture with 3T3-L1 supernatant induced by 0 and 100ng/ml LAMC1 for 48h. 10uM DC260126 was used to inhibit the effect of palmitic acid. **(C)** The RT-qPCR and Western blots were used for analyzing transfection efficiency in gastric cancer cell lines infected with siSTAT3. **(D)** LAMC1 expression determined by RT-qPCR, Western blots in BGC823 cells transfected with siSTAT3. Error bars, SD. *P < 0.05; **P < 0.01; ***P < 0.001.

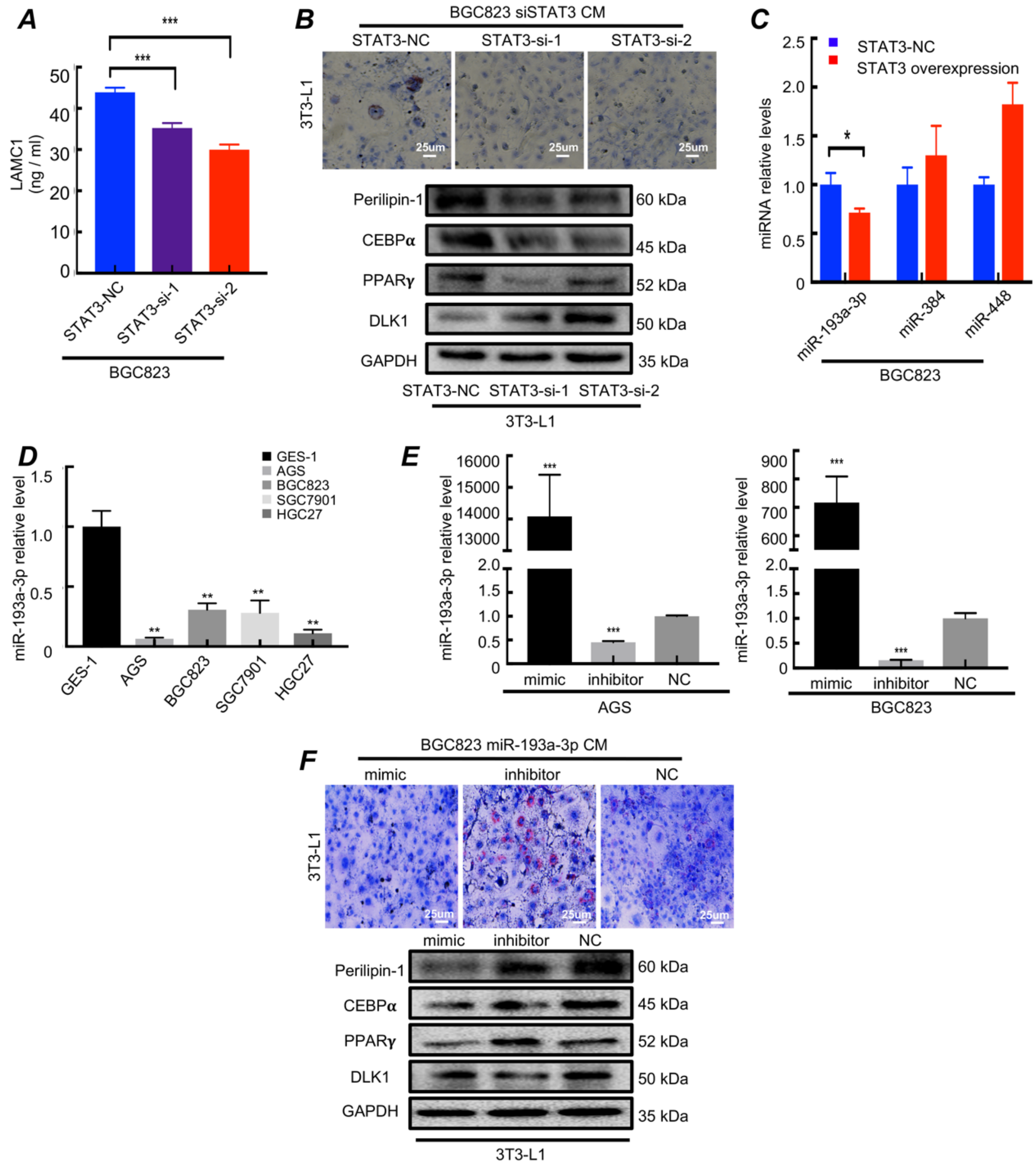

Supplementary Fig. 5 (A) ELISA for analyzing LAMC1 expression in supernatant in BGC823 cells transfected with siSTAT3. **(B)** The gastric cancer cell supernatant transfected with siSTAT3 for 48h was collected to coculture with 3T3-L1 for 5 days. The Oil Red O staining and Western blots were used for lipid formation ability. **(C)** RT-qPCR was for analyzing miR193a-3p, miR384 and miR448 expression levels in BGC823 cells with STAT3 overexpression. **(D)** The expression of miR-193a-3p was analyzed by RT-qPCR in gastric cancer cell lines. **(E)** The RT-qPCR was used for analyzing transfection efficiency in gastric cancer cell lines infected with miR-193a-3p mimic, inhibitor or NC. **(F)** The gastric cancer cell supernatant transfected with miR-193a-3p mimic, inhibitor or NC for 48h was collected to coculture with 3T3-L1 for 5 days. The Oil Red O staining and Western blots were used for lipid formation ability. Error bars, SD. *P < 0.05; **P < 0.01; ***P < 0.001.

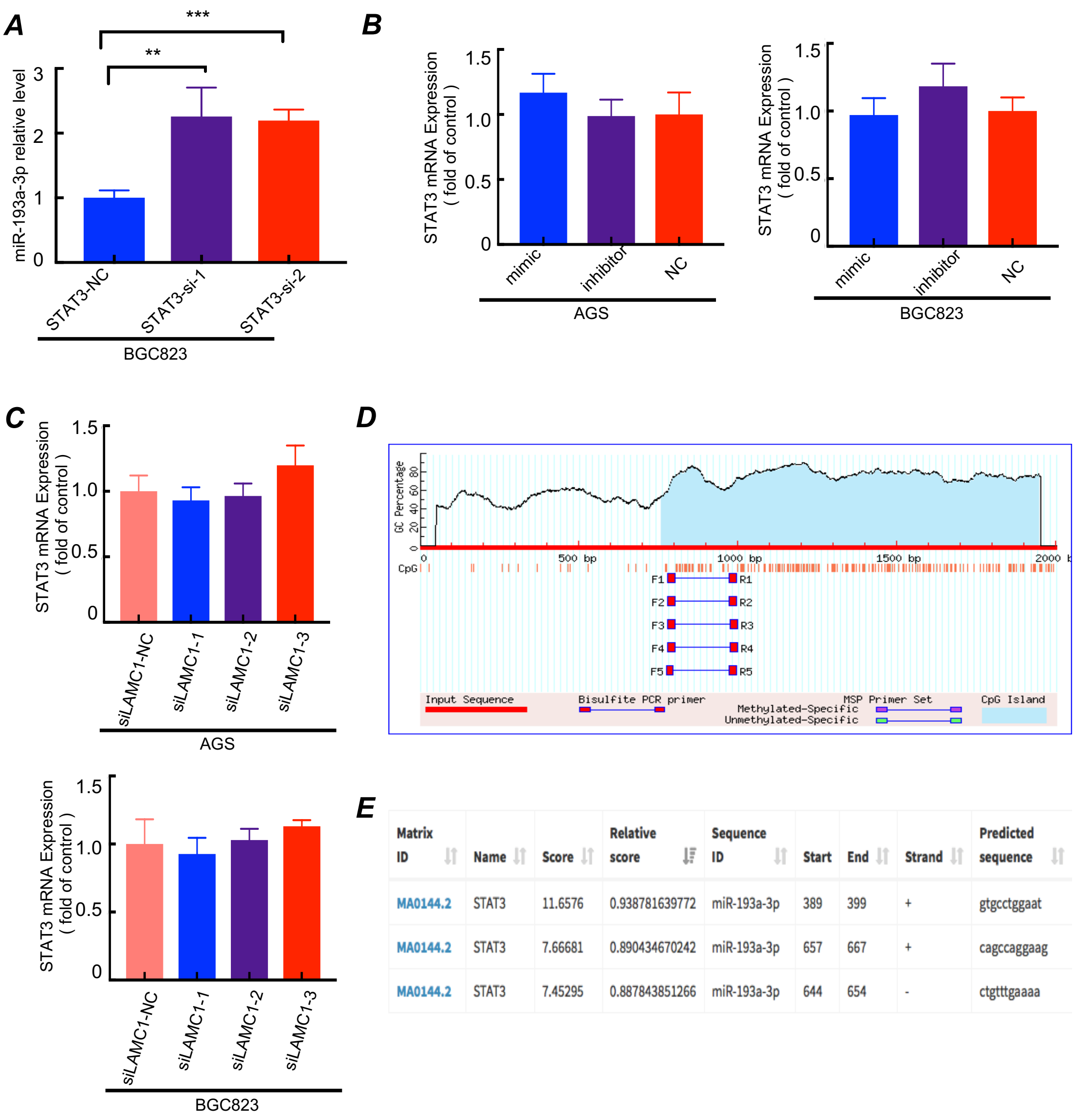

Matrix ID	Name	Score	Relative score	Sequence ID	Start	End	Strand	Predicted sequence
MA0144.2	STAT3	11.6576	0.938781639772	miR-193a-3p	389	399	+	gtgcctggaat
MA0144.2	STAT3	7.66681	0.890434670242	miR-193a-3p	657	667	+	cagccaggaag
MA0144.2	STAT3	7.45295	0.887843851266	miR-193a-3p	644	654	-	ctgtttgaaaa

Supplementary Fig. 6 (A) BGC823 cells transfected with siSTAT3 had a high miR-193a-3p expression determined by RT-qPCR. **(B-C)** The gastric cancer cells transfected with siLAMC1, miR-193a-3p mimic, inhibitor or mimic NC had no effects on STAT3 expression. **(D)** The CpG island region predicted in MethPrimer database of miR-193a-3p promoter was shown. **(E)** The promoter sequences that might be related with STAT3-mediated miR-193a-3p regulation were predicted in Jaspar database with relative profile score threshold > 85%. Error bars, SD. *P < 0.05; **P < 0.01; ***P < 0.001.

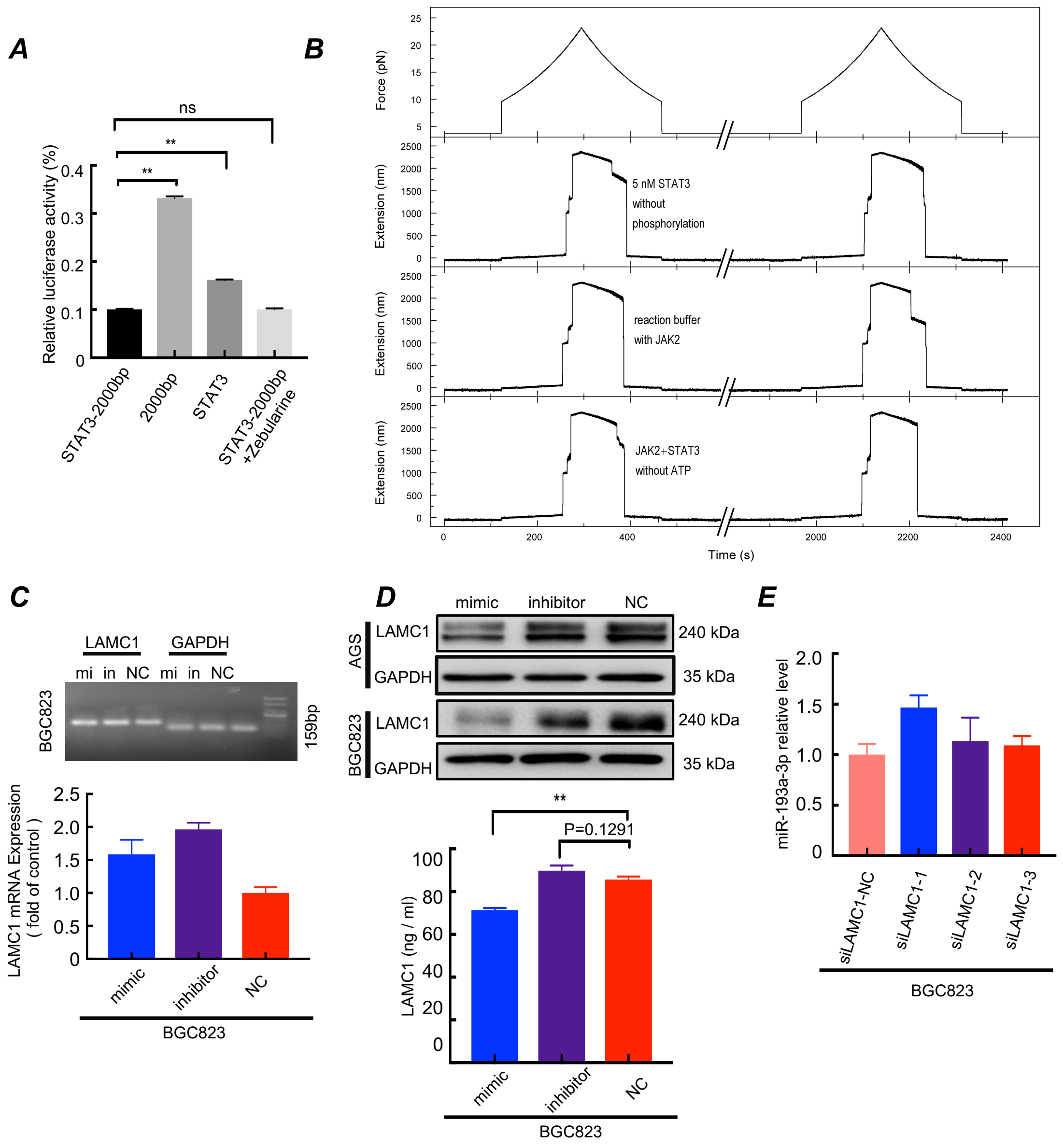

Supplementary Fig. 7 (A) The PGL4 plasmids carrying miR-193a-3p promoter constructs and PCDNA3.1 vector containing STAT3 were transfected in 293T cells with or without zebularine (50uM), and the relative luciferase activity was measured after 24h. **(B)** Force vs. time and DNA extension vs. time curve for promoter of miR-193a-3p in different conditions. **(C-D)** BGC823 cells were transfected with miR-193a-3p mimic, inhibitor or NC. The agarose gel electrophoresis and RT-qPCR were used to measure RNA level change, Western blots and Elisa for protein level. **(E)** The BGC823 cells were transfected with siLAMC1, RT-qPCR for miR-193a-3p expression. Error bars, SD. *P < 0.05; **P < 0.01; ***P < 0.001.

Table S1 90 pairs of tissue sample information

Case	Gender	Age	CEA (ng/ml)	CA125 (U/ml)	CA199 (U/ml)	T stage	N stage	TNM stage	LAMC1 expression
1	male	59	1.27	5.76	4.63	T4	N3	III	high
2	male	70	2.75	7.74	15.88	T1	N0	I	high
3	female	57	0.58	8.55	5.49	T4	N2	III	high
4	female	81	4.39	30.64	5.73	T3	N1	II	high
5	male	82	3.59	26.17	4.91	T3	N2	III	high
6	male	67	2.7	10.64	7.07	T2	N2	II	high
7	female	47	0.31	29.99	6.36	T4	N3	III	high
8	female	72	2.63	5.9	7.46	T4	N1	III	high
9	female	77	15.98	15.6	59.12	T4	N1	III	high
10	male	74	2.2	47.46	27.21	T4	N2	III	high
11	male	60	1.25	8.9	<2	T3	N1	III	high
12	male	50	1.64	5.15	17.15	T4	N1	III	high
13	female	78	1.67	9.71	9.07	T4	N2	III	high
14	male	53	1.28	20.9	4.96	T4	N2	III	high
15	male	59	2.66	14.14	212.9	T4	N0	II	high
16	male	52	5.04	9.07	10.14	T4	N3	II	high
17	female	63	4.83	18.8	22.05	T4	N3	III	high
18	male	70	3.9	6.49	23.08	T4	N0	II	high
19	female	60	2	9	7.55	T4	N0	II	high
20	female	78	2.59	8.15	95.08	T2	N3	III	high
21	male	60	1.34	13.8	4.55	T4	N1	III	high
22	male	45	2.72	38.13	219.1	T4	N3	III	high
23	male	61	131.9	9.39	36.7	T4	N3	III	high
24	male	68	2.55	25.22	4.58	T4	N3	III	high
25	male	83	2.23	8.61	>1000	T4	N3	III	high
26	male	44	2	8.8	21.72	T4	N0	II	high
27	male	59	2.55	7.97	12.85	T4	N0	III	high
28	male	55	3.1	10.75	10.03	T4	N0	III	high
29	male	69	2.03	5	125.5	T3	N0	II	high
30	female	83	1.64	14.18	26.69	T4	N1	III	high
31	male	62	0.61	8.58	5.02	T4	N2	III	high
32	female	62	0.9	10.3	8.46	T3	N1	II	high
33	male	56	1.96	16.39	3.29	T4	N0	III	high
34	male	74	1.64	6.73	4.14	T3	N3	III	high
35	female	67	2.9	18.87	8.17	T3	N3	III	high
36	female	73	1.61	6.07	0.86	T4	N3	III	high
37	female	60	1.32	12.25	18.34	T4	N3	III	high
38	male	66	7.04	55.6	3.55	T4	N1	III	high
39	male	67	763.4	270.3	>1000	T4	N1	III	high
40	female	58	2.08	4.76	24.38	T4	N2	III	high
41	male	55	0.99	6.34	2.93	T4	N1	III	high

42	male	66	0.59	11.48	3.81	T4	N0	III	high
43	female	56	0.9	4.76	9.06	T3	N3	III	high
44	male	62	0.9	4.76	9.06	T1	N0	I	high
45	female	56	20.3	10.39	11.93	T1	N1	I	high
46	female	68	4.21	6.85	19.68	T4	N3	III	low
47	male	52	0.63	8.42	0.3	T4	N2	III	low
48	male	26	1.6	6.84	33.72	T1	N0	I	low
**49**	male	58	1.34	18.7	6.13	T4	N3	III	low
50	male	65	1.02	7.51	9.12	T1	N0	I	low
51	female	57	0.54	85.22	5.61	T2	N2	II	low
52	male	76	2.13	6.11	13.8	T4	N2	III	low
53	male	62	6.59	19.8	196.68	T4	N1	III	low
54	male	32	1.16	4.9	7.51	T4	N2	III	low
55	male	78	1.92	17.27	19.69	T4	N1	III	low
56	male	77	2.9	33.8	22.22	T3	N3	III	low
57	female	54	1.28	8.8	2.03	T4	N0	II	low
58	male	74	11	94.93	2.12	T4	N3	III	low
59	male	71	1.83	10.77	64.11	T4	N2	III	low
60	female	71	0.71	13.61	9.37	T4	N0	II	low
61	male	57	1.81	9.05	<0.3	T1	N0	I	low
62	male	63	1.98	5.39	1.78	T1	N0	I	low
63	male	61	1.88	10.53	11.57	T4	N2	III	low
64	male	57	3.79	14.29	6.45	T2	N0	I	low
65	male	68	4.72	14.6	22.51	T3	N2	III	low
66	male	65	2.78	7.24	3.08	T1	N0	I	low
67	female	45	0.98	28.04	3.53	T4	N3	III	low
68	male	73	1.51	23.44	2.54	T4	N3	III	low
69	female	52	0.77	9.19	16.24	T3	N3	III	low
70	male	62	2.49	7.95	2.71	T2	N0	I	low
71	male	63	4.96	5.69	131.7	T3	N0	II	low
72	male	71	2.9	15.7	860.09	T4	N1	III	low
73	male	60	6.97	23	16.57	T2	N2	II	low
74	male	69	1.83	4.23	8.24	T4	N1	III	low
75	male	70	2.87	5.66	19.75	T4	N0	II	low
76	male	53	5.4	10.19	15.14	T4	N3	III	low
77	female	68	1.92	5.31	9.27	T2	N0	I	low
78	male	52	4.82	9.4	3.22	T4	N0	II	low
79	male	63	1.8	14.67	5.31	T4	N0	II	low
80	male	49	1.08	5.33	8.09	T1	N0	I	low
81	male	40	1.98	28.29	11.04	T1	N0	I	low
82	male	35	2.18	6.41	5.56	T4	N3	III	low
83	male	70	1.66	9.2	8.55	T4	N3	III	low
84	female	58	2.25	5	9.62	T4	N3	III	low
85	female	56	2.02	4.6	15.92	T2	N0	I	low

86	male	49	2.33	51.19	4.28	T1	N0	I	low
87	male	69	3.33	11.7	<0.30	T3	N1	II	low
88	male	46	2.57	10.1	2.21	T1	N0	I	low
89	male	48	0.69	5.42	7.91	T4	N2	III	low
90	male	64	1.62	6.6	6.03	T4	N0	II	low

Table S2 Three specimens of gastric cancer peritoneal metastasis sites

Patient	Age	Gender	Pathological type	Stage	Mass spectrometry
case 1	70	female	gastric adenocarcinoma	pT4aN1M1	✓
case 2	64	male	gastric adenocarcinoma	pT4N3bM1	✓
case 3	61	female	gastric adenocarcinoma	pT4N3M1	

Table S3 Primer sequence

Gene	Primer	Sequences (5′ to 3′)
SPARC	FORWARD	GCAGCAATGACAACAAGACCTTCG
	REVERSE	TCAGCTCAGAGTCCAGGCAAGG
COL1A1	FORWARD	GCGAGAGCATGACCGATGGATTC
	REVERSE	GCCTTCTTGAGGTTGCCAGTCTG
CCN2	FORWARD	ACCTGTGCCTGCCATTACAACTG
	REVERSE	GCCATGTCTCCGTACATCTTCCTG
THBS1	FORWARD	GGCACCAACCGCATTCCAGAG
	REVERSE	GCACAGCATCCACCAGGTCTTG
FN1	FORWARD	ATGCAACGATCAGGACACAAGGAC
	REVERSE	TGCCTCTCACACTTCCACTCTCC
LAMC1	FORWARD	GCCTTCCTGACCGACTACAACAAC
	REVERSE	GCGGCTGGTGTGGAACTTGAG
IL8	FORWARD	CTAGGCATCTTCGTCCGTCC
	REVERSE	CAGAAGCTTCATTGCCGGTG
MCP-1	FORWARD	CACTCACCTGCTGCTACTCA
	REVERSE	GCTTGGTGACAAAAACTACAGC
IP-10	FORWARD	TCTGAGTGGGACTCAAGGGAT
	REVERSE	GAGGCTCTCTGCTGTCCATC
TNFα	FORWARD	ACCCTCACACTCACAAACCA
	REVERSE	ATAGCAAATCGGCTGACGGT
IL1	FORWARD	CCTTGGAGCTATGAGTTGGACA
	REVERSE	AGCCAAGGAGACTCGGTAGA
Adiponectin	FORWARD	CTCCACCCAAGGGAACTTGT
	REVERSE	TAGGACCAAGAAGACCTGCATC
Resistin	FORWARD	CTTCCTGATGTCGGGGAAGTG
	REVERSE	TGAGTGCAGGTGCCTGTAGA
Leptin	FORWARD	GTGTCGGTTCCTGTGGCTTT
	REVERSE	GGATACCGACTGCGTGTGTG
PAI-1	FORWARD	TGCCCCACTTCTTCAAGCTC
	REVERSE	GGGGTGGTGAACTCAGTGTA
Apelin	FORWARD	TGAATCTGAGGCTCTGCGTG
	REVERSE	AACATCAGTGGCACTCCACA
STAT3	FORWARD	GCTGCCCCATACCTGAAGAC
	REVERSE	GTAGGCGCCTCAGTCGTATC
HSL	FORWARD	TCCAGGGAGCCAAATCCAAG
	REVERSE	ATGAGCCTTGAGGCTGTATCC

GAPDH	FORWARD	CTGTTCGACAGTCAGCCGCATC
	REVERSE	GCGCCCAATACGACCAAATCCG
PPAR gamma	FORWARD	GGTGACCAGAAGCCTGCATT
	REVERSE	TGTCAACCATGGTCATTTCGTT
FABP4	FORWARD	ACAGGAAAGTCAAGAGCACCAT
	REVERSE	AACTCTCGTGGAAGTGACGC
HK2	FORWARD	GTGACGCCAAAATCACGTCTC
	REVERSE	AGAGATACTGGTCAACCTTCTGC
LDHA	FORWARD	CCGCCGATTCCGGATCTCAT
	REVERSE	AGGTCAAGATATCCACTTTGCCA
GLUT1	FORWARD	TGGCATCAACGCTGTCTTCT
	REVERSE	CTAGCGCGATGGTCATGAGT
GAPDH	FORWARD	AATGGGCAGCCGTTAGGAAA
	REVERSE	GCCCAATACGACCAAATCAGAG
PKM2	FORWARD	TGTGCTACTCAGATGCTGGA
	REVERSE	GTGACTTGAGGCTCGCACAA
CPT1B	FORWARD	GTGGGTTCCTCCTTCTGCAA
	REVERSE	ACAGACTCTAGGTAAGCCCA
CD36	FORWARD	CTGAGGACTGCAGTGTAGGA
	REVERSE	TTTCTACAAGCTCTGGTTCTTATTC
hsa-miR-193a-3p	FORWARD	CGCGAACTGGCCTACAAAGT
	REVERSE	AGTGCAGGGTCCGAGGTATT
	RT Primer	GTCGTATCCAGTGCAGGGTCCGAGGTATTCGCACTGGATACGACACTGGG
hsa-miR-384	FORWARD	CGCGCGATTCCTAGAAATTG
	REVERSE	AGTGCAGGGTCCGAGGTATT
	RT Primer	GTCGTATCCAGTGCAGGGTCCGAGGTATTCGCACTGGATACGACTATGAA
hsa-miR-448	FORWARD	GCGCGTTGCATATGTAGGATG
	REVERSE	AGTGCAGGGTCCGAGGTATT
	RT Primer	GTCGTATCCAGTGCAGGGTCCGAGGTATTCGCACTGGATACGACATGGGA
U6	FORWARD	CTCGCTTCGGCAGCACA
	REVERSE	AACGCTTCACGAATTTGCGT
	RT Primer	AACGCTTCACGAATTTGCGT
